# Dynamic Expression, Differential Regulation and Functional Diversity of the CNGC Family Genes in Cotton

**DOI:** 10.3390/ijms23042041

**Published:** 2022-02-12

**Authors:** Junheng Zhao, Song Peng, Hongtu Cui, Panyu Li, Tianming Li, Luole Liu, Hanfeng Zhang, Zengyuan Tian, Haihong Shang, Ruqiang Xu

**Affiliations:** 1 School of Agricultural Sciences, Zhengzhou University, Zhengzhou 450001, China; zhaojh9605@gmail.com (J.Z.); pengsong9509@gmail.com (S.P.); dlutcuihongtu@gmail.com (H.C.); lipy0405@gmail.com (P.L.); ltm678@hotmail.com (T.L.); l1424065794@hotmail.com (L.L.); hfzhang@zzu.edu.cn (H.Z.); tianzengyuan@zzu.edu.cn (Z.T.); shh9119@163.com (H.S.); 2 Zhengzhou Research Base, State Key Laboratory of Cotton Biology, Zhengzhou University, Zhengzhou 450001, China

**Keywords:** cotton, cyclic nucleotide-gated channels, expression, regulation, cyclic AMP

## Abstract

Cyclic nucleotide-gated channels (CNGCs) constitute a family of non-selective cation channels that are primarily permeable to Ca^2+^ and activated by the direct binding of cyclic nucleotides (i.e., cAMP and cGMP) to mediate cellular signaling, both in animals and plants. Until now, our understanding of CNGCs in cotton (*Gossypium* spp.) remains poorly addressed. In the present study, we have identified 40, 41, 20, 20, and 20 CNGC genes in *G. hirsutum*, *G. barbadense*, *G. herbaceum*, *G. arboreum*, and *G. raimondii*, respectively, and demonstrated characteristics of the phylogenetic relationships, gene structures, chromosomal localization, gene duplication, and synteny. Further investigation of CNGC genes in *G. hirsutum*, named *GhCNGC1-40*, indicated that they are not only extensively expressed in various tissues and at different developmental stages, but also display diverse expression patterns in response to hormones (abscisic acid, salicylic acid, methyl jasmonate, ethylene), abiotic (salt stress) and biotic (*Verticillium dahlia* infection) stimuli, which conform with a variety of *cis*-acting regulatory elements residing in the promoter regions; moreover, a set of *GhCNGCs* are responsive to cAMP signaling during cotton fiber development. Protein–protein interactions supported the functional aspects of *GhCNGCs* in plant growth, development, and stress responses. Accordingly, the silencing of the homoeologous gene pair *GhCNGC1*&*18* and *GhCNGC12*&*31* impaired plant growth and development; however, *GhCNGC1*&*18*-silenced plants enhanced *Verticillium* wilt resistance and salt tolerance, whereas *GhCNGC12*&*31*-silenced plants had opposite effects. Together, these results unveiled the dynamic expression, differential regulation, and functional diversity of the CNGC family genes in cotton. The present work has laid the foundation for further studies and the utilization of *CNGCs* in cotton genetic improvement.

## 1. Introduction

Cyclic nucleotide-gated channels (CNGCs) are nonselective cation channels first identified in animals; they form heterotetrameric complexes consisting of two or three different types of subunits, and are opened by the direct binding of cyclic nucleotides (cNMPs; cAMP and cGMP) and modulated by Ca^2+^/calmodulin and phosphorylation; their strong permeability for Ca^2+^ provides an intracellular Ca^2+^ signal that is crucially important for both excitation and adaptation, and thus, for the channel’s function to mediate light adaptation and chemosensation, as well as playing roles in neuronal pathfinding or synaptic plasticity [1,2]. Cyclic nucleotides and Ca^2+^ are among the most well established intracellular second messenger molecules present in almost all living organisms [3]. The unique position of CNGCs as ligand-gated Ca^2+^-permeable channels suggests that they function at key sites where cNMPs and Ca^2+^ signaling pathways interact [4]. In plants, calcium is an essential nutrient, and intracellular changes in free Ca^2+^ levels act as regulators in many growth and developmental processes and coordinate responses to developmental cues and environmental stimuli [5,6]; in contrast, recent advances support that cAMP (and cGMP) constitutes an important component of the complex signaling network, including key pathways mediated by hormones, lipid, sugar, Ca^2+^, K^+^, nitrate, etc. [7,8,9,10,11,12]. Thus, it is anticipated that CNGCs may play pivotal roles during growth and development in plants.

Only 6, 4, and 6 genes encoding CNGCs have been reported in the human, *Drosophila*, and *C. elegans* genomes, respectively [1]. However, a much larger CNGC family was identified in many different plant species. A plant CNGC homologue was first identified as a calmodulin-binding transporter from barley (*Hordeum vulgare* L.) in 1998 [13]. A total of 20 CNGC homologues (AtCNGC1-20) have been identified in the genome of *Arabidopsis thaliana*, which form distinctive groups (I, II, III, IV-A and IV-B) by phylogenetic analysis [14]. Genome-wide identification using bioinformatics tools has revealed 16 OsCNGCs in rice (*Oryza sativa*) [15], 21 PbrCNGCs in pear (*Pyrus bretchneideri* Rehd.) [16], 18 SICNGCs in tomato (*Solanum lycopersicum*) [17], 26 BoCNGCs in *Brassica oleracea* [18], 30 BrCNGCs in Chinese cabbage (*Brassica rapa pekinensis*) [19], 47 TaCNGCs in wheat (*Triticum aestivum*) [20], 12 ZmCNGCs in maize (*Zea mays*) [21], 35 NtabCNGCs in tobacco (*Nicotiana tabacum*) [22], and 21 MtCNGCs in *Medicago truncatula* [23]. Like animal CNGCs, plant CNGC polypeptides have all the conserved features of a cyclic nucleotide-binding domain (CNBD) and a calmodulin binding domain (CaMBD), as well as a six transmembrane/one pore tertiary structure; however, the pore and CNBD sequences of plant CNGCs differ from animal and other plant ion channel families [24]. In addition, plant CNGCs contain overlapping CNBDs and CaMBDs, whereas CaMBD in animal CNGCs is located distal to the CNBD, near the N-terminus, implying that mechanisms for modulating protein activity have differentially evolved between animal and plant CNGCs [25]. Intriguingly, an amino acid motif that is only found in the phosphate binding cassette (PBC) and hinge regions within the CNBD of plant CNGCs has been identified, which provides an additional diagnostic tool to annotate plant CNGCs [24,25].

Until now, the roles of CNGCs in plants have been mostly elucidated in *Arabidopsis thaliana* using mutant plants. Plant CNGCs are primarily localized to the plasma membrane, but also reside in mitochondria, vacuoles, and the nucleus, where they mainly conduct Ca^2+^ flux and mediate Ca^2+^ signals [25,26]. It has been suggested that CNGCs may serve the main cNMP effectors in plant cells, sense changes in intracellular cNMP levels, and regulate numerous cellular responses [7,25]. In *Arabidopsis*, *AtCNGCs* have been implicated in various biological processes, regulating diverse aspects of growth and development, as well as biotic and abiotic stress responses, such as germination, hypocotyl elongation, gravitropism, root growth, tip growth in root hairs, leaf growth and senescence, floral transition, polarized tip growth of pollen, ion uptake and homeostasis, and responses to pathogens and herbivore attack, as well as various abiotic stresses (cold, heat, salt, drought, heavy metals) [25,27]. *CNGCs* were also identified as being involved in symbiosis in legume roots [23]. Clearly, *CNGCs* are of great interest to the community in plant breeding and genetic improvement. While the CNGC family members of numerous crop plants have been identified in recent years, as described above, their specific roles in the control of agronomic characters and crop genetic improvement remain largely unclear. Recently, studies of a dominant low seed-setting rate rice mutant (*sss1-D*) from the rice breeding program have revealed that *OsCNGC13* promotes seed-setting rate by facilitating pollen tube growth in stylar tissues [28]. The isolation and characterization of a natural rice mutant *cds1* indicated that *OsCNGC9* mediates cytoplasmic calcium elevation and positively regulates the resistance to rice blast disease; moreover, *OsCNGC9* overexpression confers enhanced rice PTI (pathogen-associated molecular pattern (PAMP)-triggered immunity) and blast resistance [29]. Thus, these findings strongly supported the notion that plant CNGCs are a very promising prospect for practical application in crop genetic improvement.

Cotton (*Gossypium* spp.) is the main source of natural fiber and has very high economic value in the world. Until now, the CNGCs family in cotton is rarely documented. In the present study, we conducted the genome-wide identification and characterization of CNGCs family genes in cotton plants, which provide the foundation for further studies of CNGC genes in cotton breeding and genetic improvement.

## 2. Results and Discussion

### 2.1. Identification and Molecular Properties of the CNGC Family Members in Cotton

The *Gossypium* genus comprises approximately 45 diploid (2n = 2X = 26) and 7 tetraploid (2n = 4X = 52) species, all of which originate from a common ancestor [30]. Currently, three diploid and two tetraploid species have been sequenced, including all four commercially domesticated species, i.e., Arabian or Levant cotton *G. herbaceum* (A_1_), Asian cotton *G. arboreum* (A_2_), Upland cotton *G. hirsutum* ((AD)_1_), and Sea-Island cotton *G. barbadense* ((AD)_2_), and the extant closest wild relative *G. raimondii* (D_5_). Among them, the two cultivated tetraploids originated in the New World from the transoceanic hybridization of an A-genome ancestor resembling *G. arboreum*, with a native D-genome ancestor resembling *G. raimondii* [31]. We conducted a genome-wide analysis and identified 40, 41, 20, 20 and 20 CNGC orthologous genes in *G. hirsutum* (named *GhCNGC1*-*40*), *G. barbadense* (*GbCNGC1-41*), *G. arboreum* (*GaCNGC1-20*), *G. herbaceum* (*GheCNGC1-20*) and *G. raimondii* (*GrCNGC1-20*), respectively. Given the notion that the rate of gene loss was higher in allotetraploid cotton [32,33], the CNGC family may be critical to cotton growth and development by retaining its size during evolution.

Detailed information of the CNGC genes in the above cotton species was provided in Tables S2–S6, including the gene ID, chromosomal location, number of amino acids (aa), protein isoelectric point (pI), composition of protein domains and motifs, and subcellular localization. For the two tetraploid species (Tables S2 and S3), the GhCNGC1-40 range from 513 to 885 aa in protein sizes, except GhCNGC4 (313 aa) and GhCNGC33 (1057 aa); in contrast, GbCNGC1-41 range from 560 to 770 aa, except GbCNGC4 (313 aa). For the three diploid species (Tables S4–S6), GaCNGC1-20 and GrCNGC1-20 have protein sizes ranging from 692 to 770 aa and 582 to 770 aa, respectively; however, GheCNGC1-20 are more variable, with six proteins (GheCNGC3, 4, 7, 11, 16 and 19) ranging from 313 to 496 aa, GheCNGC8 comprising 1445 aa, and all other proteins ranging from 517–786 aa. Thus, these results may suggest a greater functional divergence within the CNGC family in *G. herbaceum* than the other four species. Most CNGC proteins in *Gossypium* species are enriched in basic amino acids with pI >7, except five of them (GhCNGC23, GbCNGC23, GheCNGC5, GheCNGC6, and GrCNGC18) with pIs ranging from 6.20 to 6.96, suggesting that these proteins are more likely associated with cellular membranes. Accordingly, most of GhCNGC1-40 were predicted to have plasma membrane localization, except GhCNGC4 in the nucleus and GhCNGC40 in chloroplasts; similarly, almost all GbCNGC1-41 were localized in the plasma membrane with the exception of GbCNGC4 in the nucleus. Among the three diploid species, GaCNGC1-20 and GrCNGC1-20 were localized in the plasma membrane; in contrast, among GheCNGC1-20 are eight members (GheCNGC3, 6, 7, 9, 11, 13, 16, and 19) localized in chloroplasts; GheCNGC4 in mitochondrial inner membrane, GheCNGC20 in the nucleus, and the remaining 10 family members in the plasma membrane. These results agreed with the reports in other plant species, showing that most members of the CNGC family are localized to the plasma membrane, but a few members may be present in mitochondria, chloroplasts, nuclei, vacuoles, endoplasmic reticulum, or the Golgi body [15,20,21,23,25,26,34]. For example, rice OsCNGC7 and OsCNGC11 were predicted to be located in the chloroplast thylakoid membrane and mitochondrial inner membrane, respectively [15]; maize ZmCNGC13 was predicted to be located in the nucleus [21]. Among the CNGC family of wheat, TaCNGC2/3B and TaCNGC11B were predicted to be located in the chloroplast thylakoid membrane, TaCNGC15A, TaCNGC15B and TaCNGC15D in the nucleus, and TaCNGC16A in the endoplasmic reticulum or plasma membrane [20]. In *Medicago truncatula*, MtCNGC15a, MtCNGC15b, and MtCNGC15c were found at the nuclear envelope [23]. In *Arabidopsis thaliana*, AtCNGC19 was localized in the vacuole membrane, while AtCNGC20 seemed to reside in the Golgi body and may target the vacuole membrane via co-expression with AtCNGC19 [34]. Interestingly, it was noted that a considerable portion (8/20) of the CNGC family in *G. herbaceum* was predicted to target chloroplasts, and such a case for the CNGC family has not been reported in other plant species. All five *Gossypium* species in this study have evolved independently in diverse geographic regions, and it was evident that the allotetraploid formation has preceded the speciation of *G. arboreum* and *G. herbaceum* [31,32,33,35]. The CNGC family of *G. herbaceum*, a species native to the semi-arid regions of sub-Saharan Africa and Arabia, may have acquired distinctive functions relevant to chloroplast activities during evolution.

### 2.2. Phylogenetic Relationships of the CNGC Family Members in Cotton

**Figure 1:**
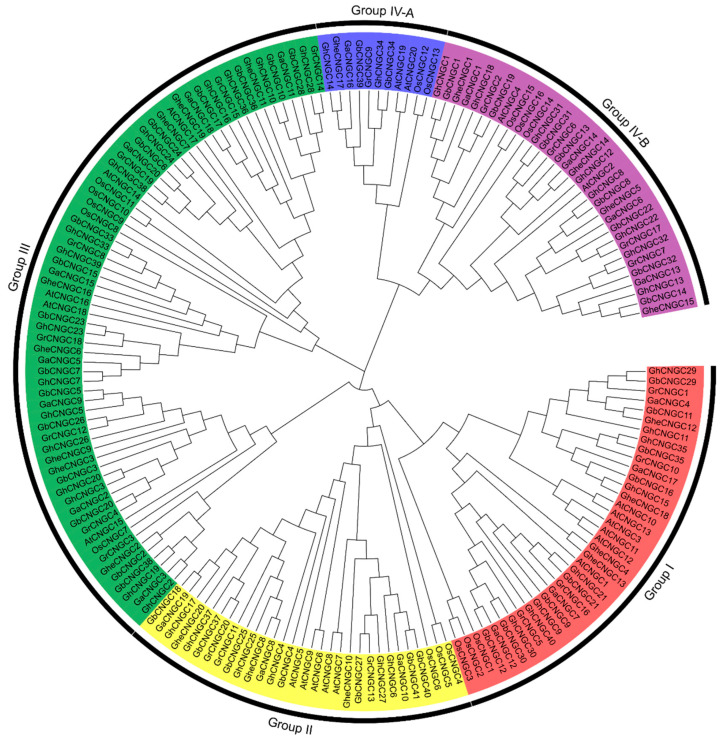
Phylogenetic relationships of the CNGC family members in cotton. The amino acid sequences of cyclic nucleotide-gated channels (CNGCs) were aligned with ClustalW, and a consensus tree was generated by the neighbor-joining (NJ) method, with 1000 bootstraps using MEGA X software [38]. The cotton CNGCs were clustered into four groups (I, II, III, IV) and two subgroups (IV-A and IV-B), conforming to the classification of CNGCs in both *Arabidopsis thaliana* and *Oryza sativa* [4,15]. *G. herbaceum*, GheCNGC1-20; *G. arboreum*, GaCNGC1-20; *G. raimondii*, GrCNGC1-20; *G. hirsutum*, GhCNGC1-40; *G. barbadense*, GbCNGC1-41; *A. thaliana*, AtCNGC1-20; *O. sativa*, OsCNGC1-16.

To obtain insights into the evolutionary relationships of CNGC family members in cotton species, a neighbor-joining phylogenetic tree was constructed, together with the CNGC family members in a representative dicot species *Arabidopsis thaliana* and a monocot species *Oryza sativa*. As shown in Figure 1, the cotton CNGCs were clearly divided into four groups (I, II, III, IV) and two distinguished subgroups (IV-A and IV-B) within group IV, conforming to the classification of the CNGC family in both Arabidopsis and rice, as reported previously [4,15]. These results confirmed the highly evolutionary conservation of the CNGC family in plants. The cotton CNGCs tended to cluster together with that from dicot species *A. thaliana*, rather than the monocot species; moreover, it was obvious that CNGCs from the five cotton species were more closely related. Thus, the CNGC family genes may have undergone apparent sequence divergence between dicot and monocot species, and they may acquire sequence differentiation between different species during evolution.

All three diploid cotton species (*G. arboreum*, *G. herbaceum* and *G. raimondii*), each containing 20 CNGC family members, have the same distribution pattern among the five phylogenetic groups, and Group IV-B contains 4 CNGC members for each species (Supplementary Figure S1); in contrast, *Arabidopsis thaliana* contains 20 CNGC genes, but Group IV-B only contains two members, indicating the diversification of the CNGC family between cotton and Arabidopsis. Both tetraploid species *G. hirsutum* and *G. barbadense* evolved from natural interspecific hybridization between two diploid species, resembling *G. arboreum* and *G. raimondii* [36,37]. Accordingly, the CNGC family members from the tetraploid tended to form 20 gene pairs from the A_t_- and D_t_-subgenome in a phylogenetic tree, exhibiting very high sequence similarity between the paired paralogous genes (Supplementary Figures S2 and S3). Similarly, the CNGC family members from *G. hirsutum* and *G. barbadense* largely formed 40 orthologous gene pairs (except GhCNGC6 versus GbCNGC40 and 41) of high sequence similarity in a phylogenetical tree (Supplementary Figure S4). These results were consistent with the notions that all the species of *Gossypium* genus originate from a common ancestor [30]; and that *G. hirsutum* and *G. barbadense* originated from a common allotetraploid ancestor and diverged recently (~0.4–0.6 million years ago) [31]. The CNGC family in the *Gossypium* genus is extremely conserved during evolution, with little expansion or contraction during the process of speciation and polyploidization, which may implicate an essential function of this protein family in cotton.

### 2.3. Chromosomal Localization, Gene Duplication, and Synteny Analysis of the CNGC Family Genes in Cotton

**Figure 2:**
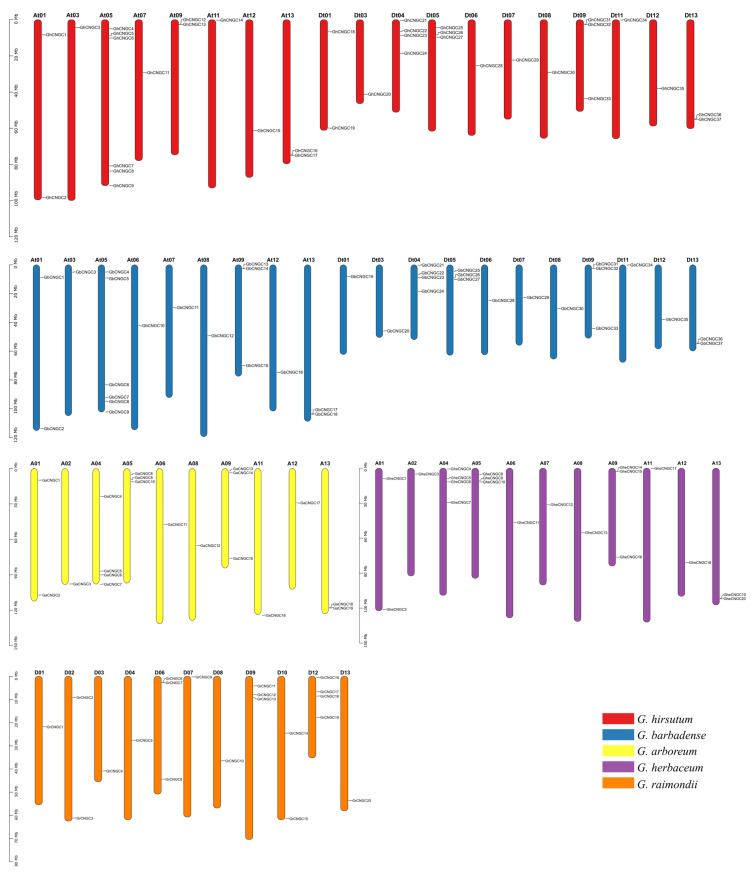
Chromosome distribution of the CNGC family genes in different cotton species. The chromosomes of five *Gossypium* species are represented by vertical bars of different colors, and the chromosome numbers are indicated at the top of each bar. The tetraploid species *G. hirsutum* and *G. barbadense* have a very similar distribution pattern of the CNGC family genes. Among the three diploid species, the distribution patterns are more similar between *G. arboreum* and *G. herbaceum*, compared to *G. raimondii*.

Chromosomal locations of the CNGC family genes in the cotton species are shown in Figure 2. The *CNGC* genes in the diploid species *G. arboreum*, *G. herbaceum*, and *G. raimondii* were located on a total of 10, 11 and 11 chromosomes, respectively, except that *GaCNGC20* resides on an unassembled scaffold; in contrast, the *CNGC* genes in the tetraploid species *G. hirsutum* and *G. barbadense* were located on a total of 19 and 20 chromosomes, respectively, except seven genes (*GhCNGC38*, *GhCNGC39*, *GhCNGC40*, *GbCNGC38*, *GbCNCG39*, *GbCNGC40* and *GbCNGC41*) on unassembled scaffolds. It was noted that *CNGC* genes in *G. hirsutum* and *G. barbadense* were distributed on the chromosomes in a highly similar pattern (Figure 2), supporting the notion that the two tetraploids recently diverged from a common allotetraploid ancestor [31]. Among the three diploids, *G. arboreum* and *G. herbaceum* shared a more similar distribution pattern of *CNGCs* on the chromosomes, compared to that in *G. raimondii*, conforming to the closer evolutionary relationship between the two domesticated species [35].

Gene families commonly arise as a result of gene duplication events, mainly including tandem, segmental, and whole-genome duplications [39,40]. The analysis of duplication events identified 6, 8, 4, 36, and 19 duplicated gene pairs in *G. arboreum*, *G. herbaceum*, *G. raimondii*, *G. hirsutum* and *G. barbadense*, respectively (Supplementary Table S7); most of them were determined as segmental duplications, except that five gene pairs (*GhCNGC12* and *GhCNGC13*; *GhCNGC31* and *GhCNGC32*; *GbCNGC13* and *GbCNGC14*; *GheCNGC14* and *GheCNGC15*; *GrCNGC6* and *GrCNGC7*) were determined as tandem duplications using MCScanX [41]. The nonsynonymous (Ka) to synonymous (Ks) substitution rate ratio (Ka/Ks) was used to serve an estimator for selective pressure on DNA sequence evolution, wherein the Ka/Ks ratio = 1, >1 and <1 implies the genes under neutral selection, positive selection, and purifying or stabilizing selection, respectively [42,43]. Consequently, the Ka/Ks ratios of *CNGC* duplications in the cotton species averaged 0.1896 ± 0.1302 (standard deviation) and ranged from 0.0319 to 0.6001, indicating that the CNGC family expansion in cotton was subjected to purifying selection, which would lead to losses of redundant genes [44]. When the Ks values were used to calculate the approximate date of duplication events using the formula t = Ks/2r, assuming the neutral substitution rate r = 2.6 × 10^−9^ substitutions/synonymous site/year for cotton [31], the divergence times of duplicated genes were estimated at least 72 million years ago (MYA) in the three diploid species (*G. arboreum*, *G. herbaceum*, *G. raimondii*) (Table S7). It is believed that the *Gossypium* genus originated from the paleo-hexaploidy of a eudicot progenitor and subsequent diversification, mainly derived from the events of diploid species divergence around 5~10 MYA and interspecific hybridization around 1~2 MYA [31,32,33,35]. Thus, the CNGC family expansion seemed to occur much earlier, likely involving the paleo-hexaploidization, than the diploid speciation divergence within *Gossypium* genus, which was well retained after allopolyploid formation. Based on a total of 338 orthologous *CNGC* pairs between the cotton species (Table S7), 97% of them had the Ka/Ks values < 1, confirming the importance of purifying selection for maintaining the functions of CNGC family genes during the evolution of these cotton species; nine gene pairs (*GaCNGC18* and *GheCNGC19*; *GhCNGC12* and *GaCNGC14*; *GhCNGC12* and *GheCNCG14*; *GhCNGC16* and *GheCNCG19*; *GhCNGC19* and *GrCNCG3*; *GbCNGC24* and *GrCNGC19*; *GhCNGC2* and *GbCNGC2*; *GhCNGC12* and *GbCNGC13*; *GhCNGC33* and *GbCNGC33*) had the Ka/Ks ratios > 1, suggesting that they were subjected to positive selection for adaptive evolution.

**Figure 3:**
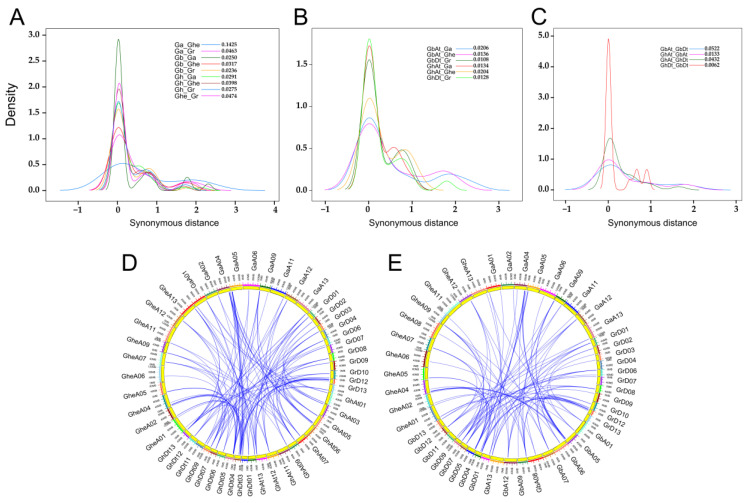
Divergence and synteny of the CNGC family genes in cotton. (**A**–**C**) Density plots of Ks distribution of the *CNGC* orthologs between the five cotton species (**A**), between the subgenomes of allotetraploid species and the corresponding genomes of three diploid species (**B**), between the subgenomes of two allotetraploid species (C); the peak positions for each comparison are indicated in the legends. (**D**,**E**) Syntenic relationships of *CNGCs* between the genomes of allotetraploid *G. hirsutum* (**D**) or *G. barbadense* (**E**) and three diploid species. Blue lines connect orthologous genes between different species. The different colored sections of the circles indicate different genomes or subgenomes. Each species is represented by their initials; Ga, *G. arboreum*; Ghe, *G. herbaceum*; Gr, *G. raimondii*; Gh, *G. hirsutum*; Gb, *G. barbadense*. At, A subgenome; Dt, D subgenome. Ks, the number of synonymous substitutions per synonymous site.

The density plots of Ks distribution peaked at 0.1425 for the *CNGC* orthologs between *G. arboreum* and *G. herbaceum*, 0.0463 between *G. arboreum* and *G. raimondii*, and 0.0474 between *G. herbaceum* and *G. raimondii*, indicating a much larger evolutionary distance of the *CNGC* orthologs between the two cultivated diploid species, which might be attributed to an impact of domestication, except for the more variable A genome compared to D genome [32]; in contrast, similar peak positions of Ks distributions were observed for both *G. hirsutum* and *G. barbadense* relative to the three diploid species (Figure 3A). However, the Ks distribution of the *CNGC* orthologs between the A_t_-subgenome of *G. hirsutum* and the genomes of *G. arboreum* and *G. herbaceum* peaked at 0.0134 and 0.0204, respectively; in contrast, the peaks appeared at 0.0206 and 0.0136 for the *CNGC* orthologs between the A_t_-subgenome of *G. barbadense* and the genomes of *G. arboreum* and *G. herbaceum*, respectively, whereas the peak positions were similar (0.0128 vs. 0.0108) for the *CNGC* orthologs between both the D_t_-subgenomes of *G. hirsutum* and *G. barbadense* and *G. raimondii* genome (Figure 3B). It has been shown that the A_t_- and D_t_-subgenome may have undergone positive selection for fiber improvement and stress tolerance traits, respectively [32,33]. Thus, the CNGC family members may potentially have involved in the differentiation of fiber traits between *G. hirsutum* and *G. barbadense*. The Ks distribution of the *CNGC* paralogs between the A_t_- and D_t_-subgenome of *G. hirsutum* shared a similar peak position (0.0432 vs. 0.0522) with that between the A_t_- and D_t_-subgenome of *G. barbadense*, which agreed with the notion that *G. hirsutum* and *G. barbadense* originated from a common allotetraploid ancestor that diverged around 0.4~0.6 MYA and independently evolved in diverse geographic regions [31]; however, the Ks distribution of the *CNGC* paralogs between the A_t_-subgenomes of *G. hirsutum* and *G. barbadense* peaked at 0.0133, in comparison with the peak at 0.0062 between the two D_t_-subgenomes, supporting the finding that sequence divergence was more common in the A_t_ subgenome than in the D_t_ subgenome [32].

The phylogenetic mechanism of CNGC family among the five cotton species was further investigated by syntenic gene analysis. Among the three diploid species, 12, 21, and 22 *CNGC* gene pairs (Table S7) showed syntenic relationships between *G. arboreum* and *G. herbaceum*, *G. arboreum* and *G. raimondii*, *G. herbaceum* and *G. raimondii*, respectively. A total of 70 syntenic *CNGC* gene pairs (Table S7) were identified between the two tetraploid species *G. hirsutum* and *G. barbadense*. There were 45, 32, and 42 syntenic *CNGC* gene pairs between *G. hirsutum* and each of the diploid species *G. arboreum*, *G. herbaceum* and *G. raimondii*, respectively (Figure 3D; Table S7); in contrast, there were 31, 31, and 32 syntenic gene pairs between *G. barbadense* and each of the diploid species *G. arboreum*, *G. herbaceum* and *G. raimondii*, respectively (Figure 3E; Table S7). In general, the CNGC family genes showed very similar patterns of syntenic relationships between the two allotetraploid species and the three diploid species (Figure 3D vs. Figure 3E), suggesting a highly conserved evolution of the CNGC family within the *Gossypium* genus.

### 2.4. Gene Structures of the CNGC Family Members in Cotton

**Figure 4:**
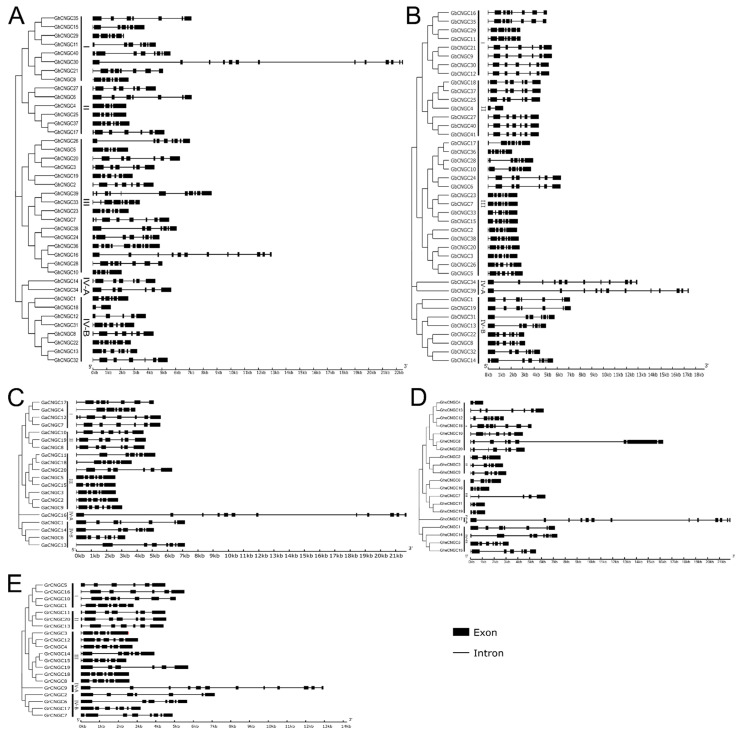
Gene structures of the CNGC family members in different cotton species. (**A**) *G. hirsutum*; (**B**) *G. barbadense*; (**C**) *G. arboreum*; (**D**) *G. herbaceum*; (**E**) *G. raimondii*. Black boxes and lines represent exons and introns, respectively. Lengths of the exons and introns are indicated by the scale at the bottom. The CNGC family members in each species are clustered into four groups (I, II, III, IV) and two subgroups (IV-A, IV-B) in a phylogenetic tree that is shown to the left in each panel.

Structural variations in genic regions and alterations in gene expression play critical roles for speciation and the evolutionary history of cotton species [31,35]. Two paralogs or orthologs were regarded as structurally divergent if they had different numbers of exons, or if they had the same number of exons, but the lengths of at least one pair of homologous exons were different [45]. The CNGC family genes in the cotton species were analyzed for structural diversity, per the exon–intron arrangement (Figure 4). For the two tetraploid species, the CNGC family genes in both *G. hirsutum* and *G. barbadense* have an average of about 7 exons, ranging from 2 to 13 exons in each gene; however, the gene structures were more diversified in *G. hirsutum* than *G. barbadense*, as evidenced by the more variable exon/intron lengths (Figure 4A vs. Figure 4B), as well as the larger coefficient of variation (CV; 27.6% vs. 22.2%) for exon numbers. For the three diploid species, the CNGC family genes in *G. arboreum*, *G. herbaceum* and *G. raimondii* each have an average of about 7, 6 and 7 exons, ranging from 6 to 12, 2 to 13, and 6 to 12 exons in each gene, respectively; the gene structures were more variable in *G. herbaceum* compared to *G. arboreum* and *G. raimondii*, per the exon/intron lengths (Figure 4D vs. Figure 4C,E) and the CVs (38.7% vs. 17.3%, 18.2%) for exon numbers. Generally, the most closely related CNGC members in the phylogenetic tree had a more similar gene structure (Figure 4), underlying their functional similarity. Intriguingly, it was noted that the phylogenetic group IV-A of the CNGC family represented the orthologous genes featuring a length much longer than other *CNGC* genes in each species except *G. hirsutum*, which featured the much longer gene *GhCNGC30*, belonging to the phylogenetic group I (Figure 4A). These *CNGC* genes of exceptionally long length may be potentially subjected to distinct regulation. It has been shown that paralogous genes, which are derived from gene duplication, and initially have identical sequences and functions, tend to diverge in regulatory and coding regions, which may result in shifts in the expression pattern and the acquisition of new functions [45]. Altogether, it was postulated that the greater gene structure variations of the CNGC family in *G. hirsutum* and *G. herbaceum* may be of significance for environmental adaptation during evolution and domestication.

### 2.5. Composition of Cis-Acting Elements in the Promoters of CNGC Genes in Upland Cotton

**Figure 5:**
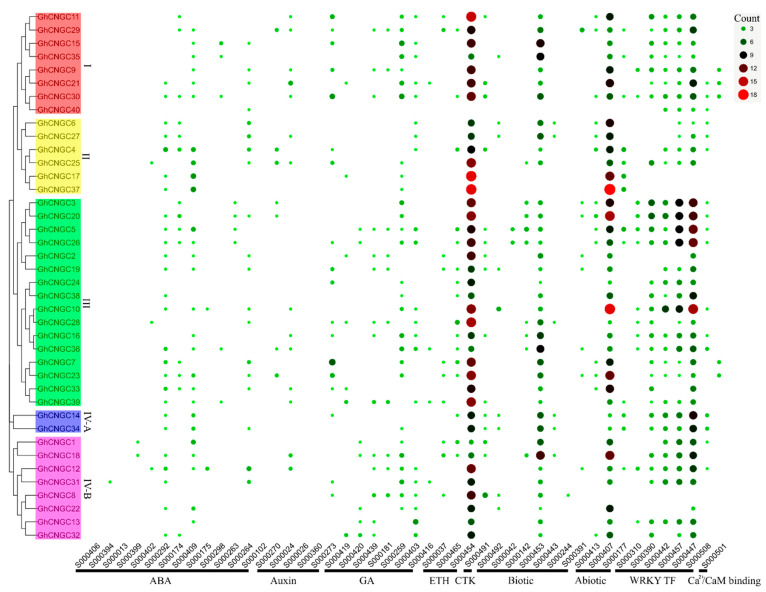
*Cis*-acting regulatory elements composition in the promoters of CNGC family genes in Upland cotton. The *cis*-acting regulatory DNA elements were determined using the PLACE database (http://dna.affrc.go.jp/PLACE/?action=newplace/ (accessed on 10 August 2021)). Each *cis*-acting element is marked by a dot with the color and size representing its count as indicated by the scale bar at the top right. Bottom side indicates the regulators and codes of *cis*-acting elements. Left side indicates the clustering groups (I, II, III, IV-A and IV-B; marked in different colors) of *GhCNGC1-40* in a phylogenetic tree. ABA, abscisic acid; GA, gibberellic acid; ETH, ethylene; CTK, cytokinin; WRKY TF, WRKY transcription factor.

The Upland cotton, *G. hirsutum*, is the predominant species for cultivation and cotton production in the world, accounting for over 90% of commercial cotton production worldwide [31]. Thus, the CNGC family genes in this species were further analyzed in the following sections. To understand the regulatory mechanisms governing the expression of *GhCNGCs*, the conserved *cis*-acting regulatory DNA elements in the promoter region of 1000 bp upstream from the translation start site for each gene (except *GhCNGC40* with an available region of 187 bp) were determined using the PLACE database [46]. Consequently, a variety of *cis*-acting elements were identified in the promoters of each *CNGC* gene (Figure 5), which are implicated in response to abscisic acid (YACGTGGC; ACGTGKC; RYACGTGGYR; CAATTATTA; ACCGAC; ACACNNG; CACATG; YAACKG; CTAACCA; TTTTTTCC; CAAACACC; CATGCA; CATGCATG), auxin (TGTCTC; TGACG; KGTCCCAT; GGTCCCAT; ACTTTA), gibberellic acid (TAACAGA; TAACGTA; TAACAAR; TAACAAA; CCTTTT; TATCCA; TATCCAC), ethylene (AWTTCAAA; TAAAATAT; NGATT), cytokinin (TATTAG), Ca^2+^/calmodulin binding (VCGCGB), WRKY transcription factor (TTTGACY; TTGAC; TGACT; TGACY; TGAC; CTGACY), biotic stress (ACCWWCC; CACGTG; TTGACC; GAAAAA; GTTAGTT; AACGTGT; YTGTCWC), and abiotic stress (CATGTG; CANNTG; TAACTG). Thus, the *GhCNGC* genes may be under the regulatory control of phytohormones, developmental cues, and environmental conditions. It was noted that most of these genes contain more copies of *cis*-acting elements related to ethylene, biotic, abiotic stresses, and WRKY transcription factor, suggesting their predominant functions likely involving environmental responses. However, the *CNGC* family genes each showed distinct patterns, per the composition and copy number of *cis*-acting elements, and even the most closely related paralogous gene pairs in the phylogenetic tree did not display an identical pattern (Figure 5), suggesting that the CNGC family members may be regulated with difference in varying degrees. In line with this notion, it was documented that the closest paralogs *AtCNGC2* and *AtCNGC4* of the CNGC family in *Arabidopsis thaliana* exhibited very similar functions using mutant plants, but also showed subtle differences in the gene-for-gene resistance response [47,48].

### 2.6. Functional Protein Association Network of the CNGC Family in Upland Cotton

**Figure 6:**
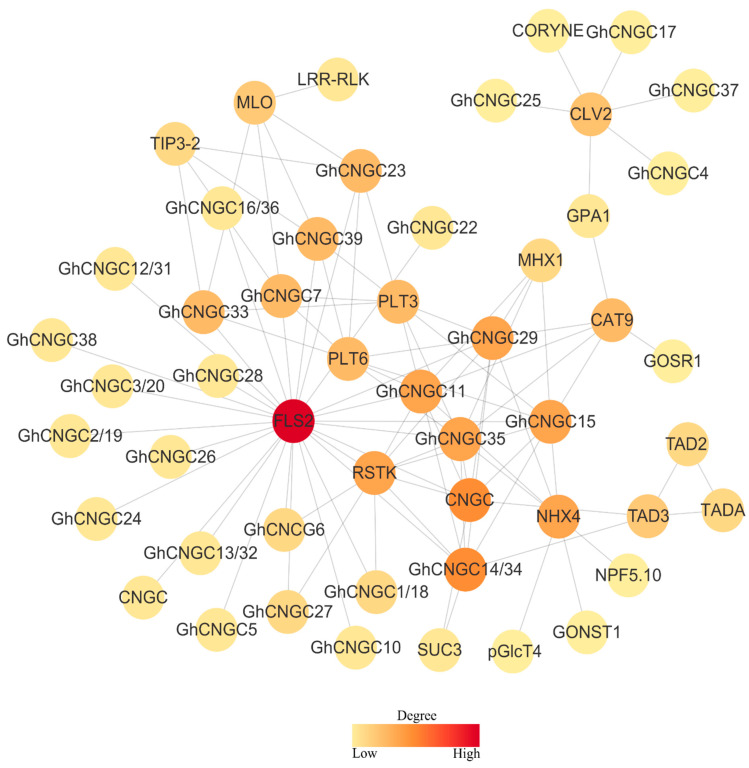
Functional interaction network of the CNGC family in Upland cotton. Nodes indicate the associated proteins, and edges represent pairwise interactions determined using STRING program (https://string-db.org/ (accessed on 26 August 2021)). Node color indicates the interaction degree as justified by the color scale bar at the bottom. The network was depicted using Cytoscape (https://cytoscape.org/ (accessed on 26 August 2021)).

The regulatory mechanisms of CNGC family genes in Upland cotton were further explored by analyzing protein interactions using the STRING database at the highest confidence score [49], resulting in the functional protein association network (Figure 6). A total of 27 GhCNGCs were shown to have a strong interaction with FLS2 (FLAGELLIN SENSING 2), which is an important regulatory receptor kinase at the plasma membrane to activate immune signaling [50]; 7 GhCNGCs (GhCNGC1, 14, 15, 18, 27, 34, 35) interacted with RSTK (receptor serine/threonine kinases), which play a central role in signaling during pathogen recognition [51]; additionally, 4 GhCNGCs (GhCNGC7, 23, 33, 39) interacted with MOL (mildew resistance locus O), which modulates plant disease defense and cell death [52]. Thus, these results may suggest a prominent role of GhCNGCs in plant immune responses. However, both RSTK and MOL have been implicated in morphological and developmental control [51,52], whereas 4 GhCNGCs (GhCNGC4, 17, 25, 37) interacted with CLV2 (CLAVATA2), which is involved in the regulation of SAM (shoot apical meristem) and RAM (root apical meristem) maintenance, affects organ development, and functions in plant–microbe interactions [53]; additionally, GhCNGC14 and GhCNGC34 interacted with TAD3 (tRNA-specific adenosine deaminase) which is essential for embryo development, and impacts plant growth [54]. Clearly, GhCNGCs may be critical players during growth and development. Among other interaction partners of GhCNGCs were a set of transporter or exchanger proteins: CAT9 (CATIONIC AMINO ACID TRANSPORTER 9) mediates cellular nitrogen-dependent amino acid homeostasis [55]; SUC3 (sucrose transporter 3) conducts energy-dependent sucrose/maltose transport, and plays a role in the sucrose import into sink tissues, as well as in the generation of osmotic gradients [56,57]. Both PLT3 and PLT6 (polyol/monosaccharide transporters) are implicated in response to environmental stimuli [58], and PLT6 is induced upon endogenous cAMP elevation [7]; NHX4 (sodium hydrogen exchanger 4) is critical for the maintenance of cellular cation homeostasis, and contributes to growth and development, as well as mediating plant stress acclimation [59,60]; MHX1 (magnesium/proton exchanger 1) is a vacuolar transporter that is important for mediating the adequate homeostasis of several divalent metal cations (i.e., Mg^2+^, Zn^2+^, Fe^2+^), which are required for many enzymatic reactions, and has been found to affect the proton homeostasis of cells and plant growth [61,62]; additionally, TIP3-2 (tonoplast intrinsic protein) is implicated in the transport of water and small neutral substrates such as urea, ammonia, and hydrogen peroxide (H_2_O_2_), and acts to modulate the response to abscisic acid (ABA) and maintain seed longevity under the control of ABI3 (ABSCISIC ACID INSENSITIVE 3), as well as affecting seed dormancy and germination in response to stress [63,64]. No doubt, the linkages of GhCNGCs with the above genes of various transport activities may be of significance for modulating plant responses to genetic and environmental stimuli. Finally, GhCNGC11, 15, 29, and 35 interacted with GhCNGC14 and 34. Recent studies have clearly shown that plant CNGCs may form both homomeric and heteromeric channels via dynamic interactions [65,66,67]. Thus, dynamic interactions between GhCNGC family members may greatly contribute to their functional diversity and regulatory complexity. 

### 2.7. Expression Profiles of the CNGC Family Genes during Growth and Development of Upland Cotton

**Figure 7:**
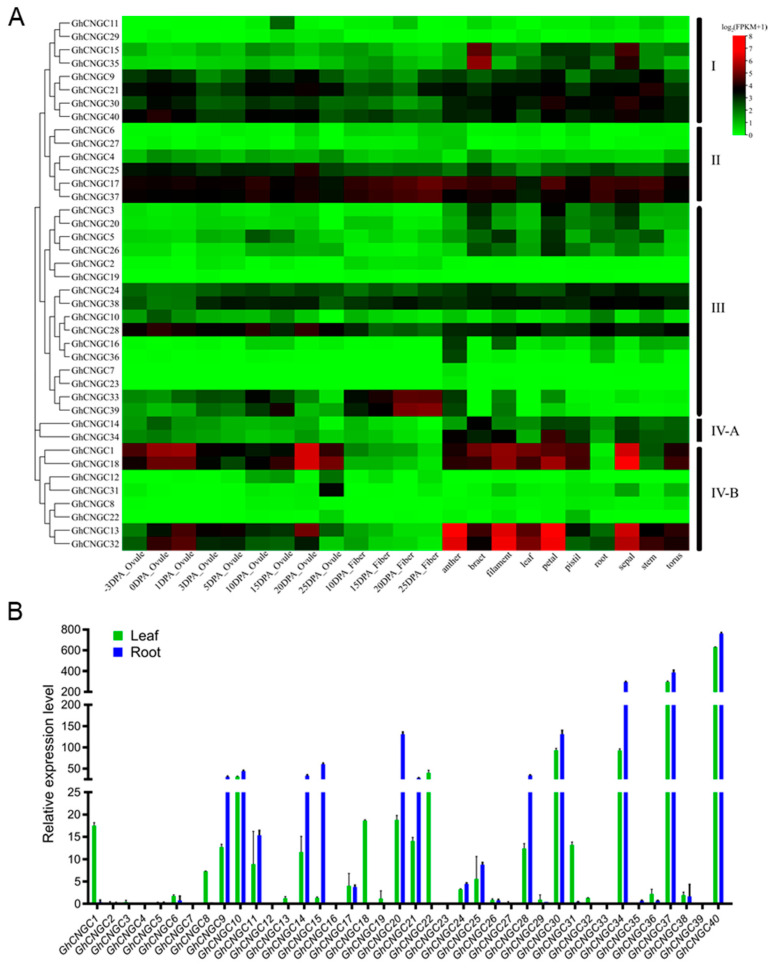
Expression profiles of *GhCNGCs* during growth and development of Upland cotton. (**A**) Heatmap depicting expression levels of *GhCNGCs* across different tissues and developmental stages. The expression levels were estimated by FPKM (fragments per kilobase of exon model per million mapped fragments) from transcriptome sequencing data [31]. DPA, days post anthesis. (**B**) Quantitative RT-PCR detection of *GhCNGCs* expression in leaves and roots. True leaves of 19-day-old plants and roots of 25-day-old plants were sampled. Relative expression levels were normalized to house-keeping gene *GhUBQ7* and calculated using the 2^−∆∆Cq^ method. Data are mean ± SD (*n* = 3).

The expression profiles of the CNGC family genes in Upland cotton were investigated across different tissues and developmental stages using the estimates of FPKM (fragments per kilobase of exon model per million mapped fragments) from transcriptome sequencing data [31]. As shown in Figure 7A, these genes were expressed at varying levels across different tissues (root, stem, leaf, anther, petal, pistil, filament, bract, sepal, torus, ovule, and fiber) and developmental stages (−3 to 25 days post anthesis (DPA)); of them, *GhCNGC1*, *9*, *13*, *17*, *18*, *21*, *28*, *30*, *32*, *37* and *40* showed higher expression levels in most tissues and at various developmental stages. Thus, GhCNGCs may function during the life cycle. These genes exhibited varying expression patterns between them, which may suggest functional differentiation. Generally, most of the highly expressed *GhCNGCs* showed higher levels in reproductive tissues than vegetative tissues, implicating that *GhCNGCs* may be critical to cotton reproduction. For example, *GhCNGC1*, *13*, *18*, and *32* were expressed most highly in ovules (0, 1, 20 DPA), anther, filament, petal and sepal; their closest homologs are *AtCNGC2* and *AtCNGC4* in Arabidopsis (Figure 1), which play important functions in the regulation of floral transition [66,68], pollen growth [69], thermal sensing and acquired thermotolerance [66], senescence and programmed cell death [70,71,72], and innate immune response [65,73]. Interestingly, *GhCNGC17*, *33*, *37*, and *39* increased expression levels during the developmental stage from 10 to 25 DPA (Figure 7A), likely contributing to fiber development. Cotton fibers are highly elongated and thickened single seed epidermal cells, resembling the tip-growing cells, such as pollen tubes and root hairs [74]. In Arabidopsis, *AtCNGC5*, the closest homolog of *GhCNGC17* and *37*, is essential for constitutive root hair growth [75]; in contrast, *AtCNGC16*, the closest homolog of *GhCNGC33* and *39*, is critical for pollen tube growth and fertility, and has been suggested to specifically impact cell walls or membrane dynamics [76].

We performed quantitative RT-PCR (qRT-PCR) to confirm the expression of *GhCNGCs* using leaf and root tissues (Figure 7B). The results indicated the relative higher expression of *GhCNGC1*, *10*, *18*, *20*, *22*, *30*, *34*, *37* and *40* in leaves, whereas *GhCNGC9*, *10*, *14*, *15*, *20*, *21*, *28*, *29*, *34*, *37* and *40* exhibited higher expression levels in roots. The relative expression levels of *GhCNGCs* in both leaf and root tissues detected by qRT-PCR were largely in agreement with the FPKM estimates by transcriptome sequencing (Figure 7A), despite some differences of the tested tissues in genotypes, growth stages, and conditions.

### 2.8. Hormonal Control of GhCNGCs Expression during Seedling Growth of Upland Cotton

**Figure 8:**
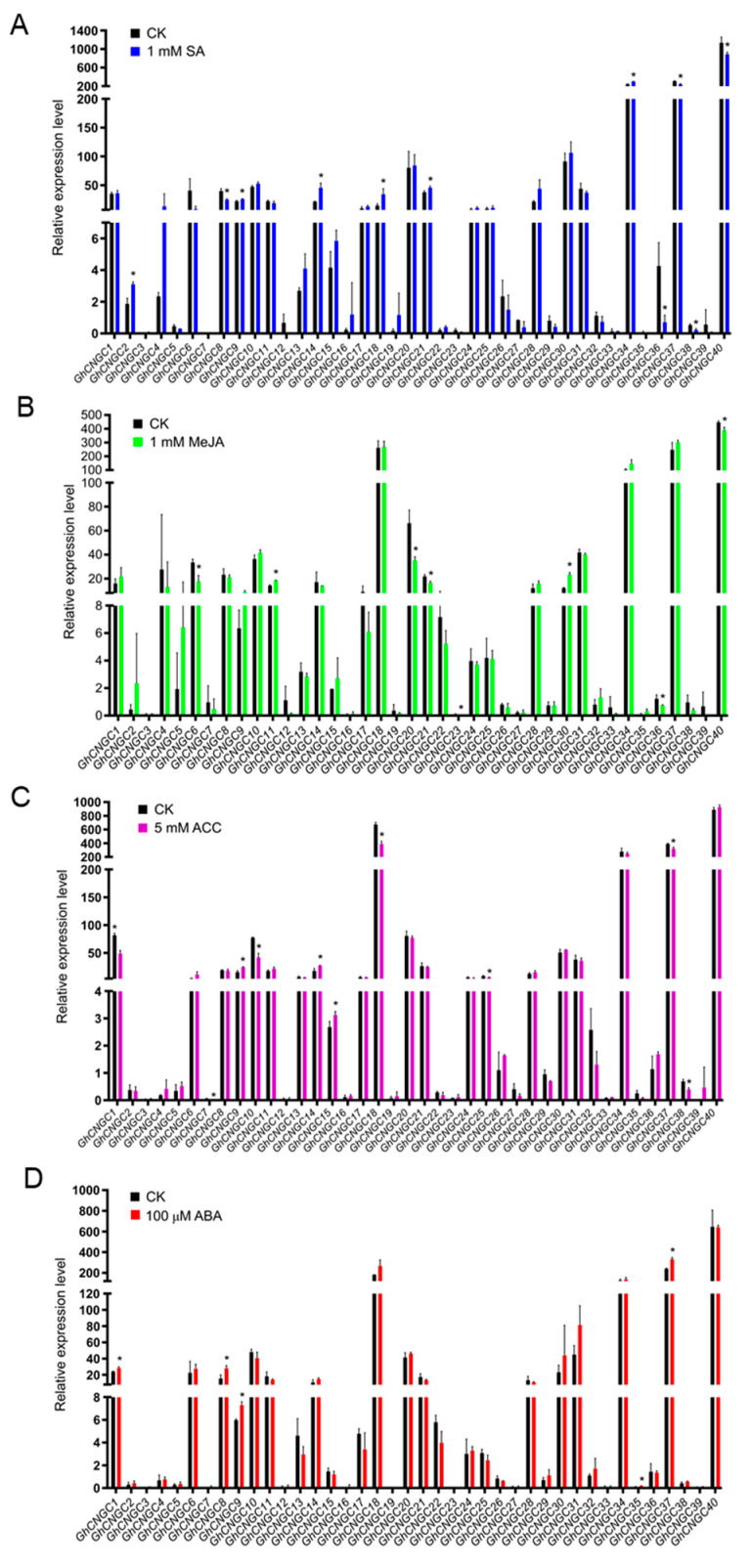
Expression of *GhCNGCs* under hormonal treatments during seedling growth of Upland cotton. (**A**) 1 mM salicylic acid (SA); (**B**) 1 mM methyl jasmonate (MeJA); (**C**) 5 mM 1-aminocyclopropane-1-carboxylic acid (ACC); (**D**) 100 µM abscisic acid (ABA). Three-week-old seedlings of *G. hirsutum* “0–153” were sprayed with water solution containing the phytohormone or mock control (CK), and the above-ground tissue samples were collected after 24 h treatment for quantitative RT-PCR detection. Relative expression levels were normalized to house-keeping gene *GhUBQ7* and calculated using the 2^−∆∆Cq^ method. Data are mean ± SD (*n* = 3), two-tailed Student’s *t*-test * *p* < 0.05.

Given the above finding that the promoters of *GhCNGCs* contain a variety of *cis*-acting regulatory elements responding to various phytohormones (Figure 5), we examined the expression of *GhCNGCs* under hormonal treatments during seedling growth. The foliar application of SA (salicylic acid) significantly upregulated *GhCNGC2*, *9*, *14*, *18*, *21* and *34*, but also down-regulated *GhCNGC8*, *36*, *37*, *38* and *40* (Figure 8A). MeJA (methyl jasmonate) treatment caused the significant up-regulation of *GhCNGC11* and *30*, as well as the obvious down-regulation of *GhCNGC6*, *20*, *21*, *23*, *36* and *40* (Figure 8B). The foliar application of ACC (1-aminocyclopropane-1-carboxylic acid), an immediate precursor of ethylene, resulted in the significant elevation of *GhCNGC9*, *14* and *15*, but apparently suppressed *GhCNGC1*, *7*, *18*, *25*, *37* and *38* (Figure 8C). ABA (abscisic acid) treatment increased the expression levels of *GhCNGC1*, *8*, *9*, *35* and *37* (Figure 8D). These results confirmed that many *GhCNGCs* were highly responsive to phytohormones; moreover, some of them (*GhCNGC1*, *8*, *9*, *14*, *21*, *36*, *37*, *38* and *40*) responded to different phytohormones, which may suggest the important roles of these genes in the coordination of the hormone signaling network. Similarly, it has been previously reported that the CNGC family genes showed significant responses to exogenously applied hormones in rice [15] and wheat [20]. More recently, it was demonstrated that *AtCNGC5*, *6* and *9* are involved in the auxin signaling of root hairs in Arabidopsis [75].

### 2.9. Regulation of GhCNGCs Expression under Abiotic and Biotic Stresses in Upland Cotton

**Figure 9:**
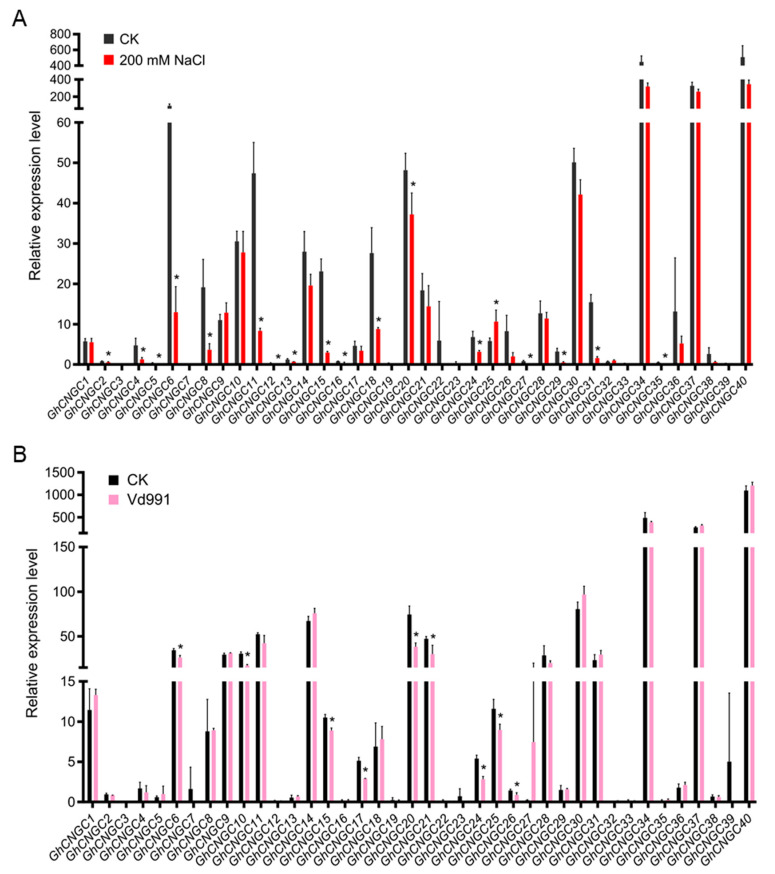
Regulation of *GhCNGCs* expression under conditions of salt stress and fungal infection in Upland cotton. (**A**) Salt stress. Cotton seeds (*G. hirsutum* “0–153”) were germinated for three days before growing with Hoagland’s nutrient solution containing 200 mM NaCl for two weeks. (**B**) Fungal infection. About 3-week-old seedlings (*G. hirsutum* “Han 8266”) were inoculated for three days with *Verticillium dahliae* stain Vd991 by the root-dipping method. Whole plant samples were collected for quantitative RT-PCR detection. Relative expression levels were normalized to house-keeping gene *GhUBQ7* and calculated using the 2^−∆∆Cq^ method. Data are mean ± SD (*n* = 3), two-tailed Student’s *t*-test * *p* < 0.05.

CNGCs have been found to play extensive functions in responses to salt, drought, cold, heat, and heavy metal stresses, as well as pathogen infection in Arabidopsis [25]. We investigated expression profiles of *GhCNGCs* under the treatment of salt stress (200 mM NaCl), indicating that almost half of them showed the significant alteration of expression levels, including the upregulation of *GhCNGC25* and the downregulation of 17 genes (*GhCNGC2*, *4*, *5*, *6*, *8*, *11*, *12*, *13*, *15*, *16*, *18*, *20*, *24*, *27*, *29*, *30* and *35*) (Figure 9A). *GhCNGC4* and *25* are the paralogous gene pair from the A- and D-subgenome of *G. hirsutum*, respectively; they showed the closest homology with *AtCNGC5* (Figure 1), which is required for constitutive root hair growth in Arabidopsis [75]; intriguingly, they were regulated in an opposite direction under salt stress. In Arabidopsis, both *AtCNGC19* and *20* were induced in response to salt stress [77], whereas *AtCNGC10* was dramatically inhibited after exposure to 200 mM NaCl, and it negatively regulated salt tolerance by mediating Na^+^ transport [78]. *GhCNGC11*, *15*, *29* and *35* seemed to exhibit similar salt stress responses to their closest ortholog *AtCNGC10*; however, this phenomenon was not observed with *GhCNGC14* and *34*, which are the closest orthologs of *AtCNGC19* and *20*. Thus, the orthologous genes of the CNGC family members in different plant species may have evolved to play species-specific roles.

Expression profiles of *GhCNGCs* were also studied in young seedlings by inoculation with *Verticillium dahliae* strain Vd991, an isolate from *G. hirsutum* [79]. Consequently, the fungal infection caused the obvious suppression of *GhCNGC6*, *10*, *15*, *17*, *20*, *21*, *24*, *25* and *26.* At least four members of the CNGCs family in Arabidopsis have been demonstrated to play critical roles in pathogen defense responses, including *AtCNGC2*, *4*, *11* and *12*. Loss-of-function mutants of these Arabidopsis genes displayed impaired hypersensitive response (HR), the constitutive expression of SA, changes in the expression pattern of pathogenesis-related (PR) genes, and the alteration of plant responses to avirulent pathogens [27]. Like *AtCNGC11* and *12*, *GhCNGC15* and *21* belong to group I in the phylogenetic tree (Figure 1), which may support similar functions between them in disease resistance; however, the corresponding *G. hirsutum* orthologous genes of *AtCNGC2* and *4* did not show significant expression induction by Vd991 infection.

### 2.10. Modulation of GhCNGCs Expression Associated with cAMP Signaling during Cotton Fiber Development

Cotton fiber is a unique elongated cell, and its development has been well known to involve sugar metabolism, hormones, secondary metabolites, and the cytoskeleton during ovule culture [80]. Very coincidentally, we recently found that cAMP signaling is predominantly linked with these biological processes in Arabidopsis [7]. Given the notion that CNGCs are directly regulated by cAMP [4,25], we wonder if cAMP signaling might modulate *GhCNGCs* expression during ovule culture. For this, we tested the effects of a set of commonly used drugs for cAMP signaling pathway studies, including a specific inhibitor (2′,3′-dideoxyadenosine; DDA) or activator (forskolin) of adenylate cyclase, as well as the membrane permeable cAMP analog (8-Br-cAMP). Adenylate cyclase is the sole enzyme responsible for the cellular production of cAMP, which stimulates cAMP-dependent protein kinase A (PKA) to phosphorylate cAMP response-element binding-protein (CREB), and subsequently activates the transcription of a variety of target genes, resulting in multiple physiological functions [81]. Both DDA and forskolin are commonly used in biological process or pathway studies involving adenylyl cyclase activity and cAMP pool modulation, whereas 8-Br-cAMP is often used to imitate cAMP activation agents, both in plants and animals [73,82,83,84]. Based on our RNA sequencing results, 13 *GhCNGCs* showed differential expression at the threshold of an absolute value of log2(fold change) > 1 and adjusted *p* < 0.05 under treatments of DDA or forskolin, or 8-Br-cAMP during ovule culture (Table 1), which included *GhCNGC39* being the closest homolog of *AtCNGC16* that has been found to be critical for pollen tube growth by specifically impacting cell walls or membrane dynamics [76]. Basically, the DDA-mediated inhibition of adenylate cyclase activities caused the suppression of most *GhCNGCs*, except the upregulation of *GhCNGC5* and *16* under condition of higher concentration (100 µM); of them, the paralogous gene pair *GhCNGC1* and *18* were all significantly down-regulated in a dose-dependent manner (Table 1), highlighting a prominent role during fiber development. In contrast, the forskolin-mediated activation of adenylate cyclase activities seemed to up-regulate most *GhCNGCs* in a general trend, but only *GhCNGC5* and *GhCNGC26* showed significant up-regulation at the above artificially set criteria. These results may be evident for the modulation of *GhCNGCs* mediated by adenylate cyclase activities; however, the exogenous application of 8-Br-cAMP seemed to have no significant impact on *GhCNGCs* during ovule culture. Interestingly, recent advances supported the notion that adenylate cyclase activities in higher plants are embedded in multidomain proteins which usually have distinctive functions in development and environmental responses [85]. For examples, both AtKUP5 and AtKUP7 contain adenylate cyclase activities for the production of cAMP, but they are essential genes for K^+^ transport in Arabidopsis [86,87]; AtLRRAC1 possesses adenylate cyclase activity and is a leucine-rich repeat (LRR) protein implicated in immune response [88]. Thus, plant adenylate cyclase activities and cAMP production seemed to be tightly coupled with other distinctive functions for development and environmental adaptation, which may pose a great challenge to elucidating cAMP signaling in plants.

### 2.11. Functional Characterization of GhCNGC1&18 and GhCNGC12&31 in Upland Cotton

**Figure 10:**
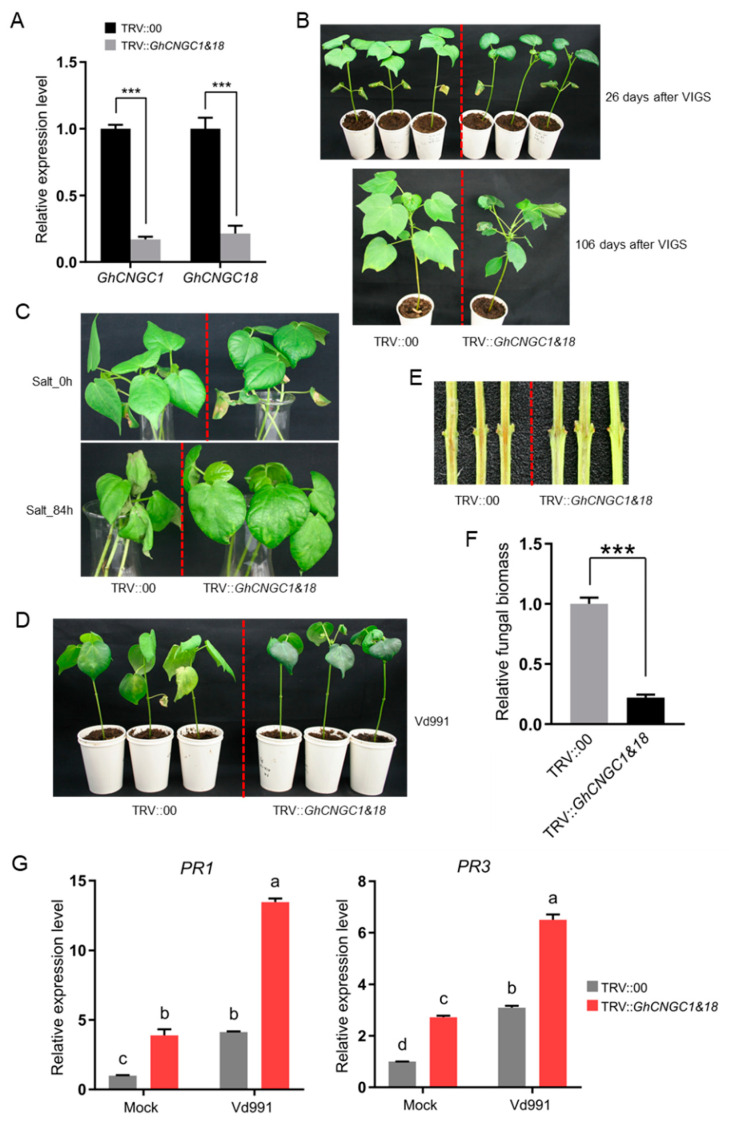
Effects of *GhCNGC1* and *GhCNGC18* simultaneous silencing in Upland cotton. Virus-induced gene silencing (VIGS) was performed by infiltration of cotyledons using *Agrobacterium* carrying the TRV::*GhCNGC1&18* vector that simultaneously targeted both *GhCNGC1* and *GhCNGC18* for silencing, or the empty vector (TRV::00) as mock control. (**A**) Relative expression levels of *GhCNGC1* and *GhCNGC18* in the silenced and mock control plants. The second true-leaf samples were used for quantitative RT-PCR detection. (**B**) Phenotypes of *GhCNGC1&18*-silenced plants versus mock control plants. The silenced plants grow smaller leaves with darker colors and downward-curled margins (top panel), as well as become stunted in stature (bottom panel). (**C**) Salt stress resistance. *GhCNGC1&18*-silenced plants show obviously enhanced salt resistance at 6 days (84 h) after 200 mM NaCl treatment, compared to mock control plants. (**D**–**F**) Fungal disease resistance. In comparison with mock control plants, *GhCNGC1&18*-silenced plants display enhanced resistance to Verticillium wilt symptoms at 16 days post inoculation (dpi) with *V. dahliae* strain Vd991 (**D**), alleviate the vascular wilt symptoms of browning in the stem tissues at 30 dpi (**E**), and reduce fungal biomass in the stem tissues at 24 dpi (**F**). (**G**) Expression of *PR* genes. The root samples for quantitative RT-PCR detection were collected from the Vd991-inoculated and mock control plants at 24 h post-inoculation. Data in (**A**,**F**,**G**) are mean ± SD (*n* = 3), Student’s *t*-test *** *p* < 0.001 for (**A**,**F**); ANOVA followed by Tukey’s multiple comparisons test for (**G**); different letters indicate significant difference at *p* < 0.05.

*Gossypium hirsutum* represents a true allotetraploid species that evolved from natural interspecific hybridization between the A- and D-genome diploid species in the New World [31,33,35]. *GhCNGCs* from the A- and D-subgenome of *G. hirsutum* formed about 20 gene pairs of very high sequence similarity in a phylogenetic tree (Supplementary Figure S2), underlying redundant functions between the paired genes. Thus, the tobacco rattle virus (TRV)-mediated virus-induced gene silencing (VIGS) assay, a robust and effective reverse genetic tool commonly used in cotton [89], was performed to simultaneously knock down the paired genes for functional analysis. For this, the silencing target fragments were designed to specifically disrupt the expression of the paired genes in the *G. hirsutum* genome. *GhCNGC1&18* are the paired genes sharing closest homology with *AtCNGC4* in Arabidopsis (Figure 1), and we obtained the expression levels reduced by 83% for *GhCNGC1* and 79% for *GhCNGC18* in the silenced plants (TRV::*GhCNGC1&18*) compared to mock control plants (TRV::00) (Figure 10A). The *GhCNGC1&18*-silenced plants grew smaller leaves with darker colors and a downward curled margin, as well as displaying stunted growth phenotype (Figure 10B). When subjected to salt stress treatment by growing in a deionized water solution containing 200 mM NaCl, *GhCNGC1&18*-silenced plants were able to maintain a largely normal growth phenotype after 6 days’ treatment, whereas mock control plants were severely withered (Figure 10C), suggesting that *GhCNGC1&18* are negatively implicated in salt stress resistance. When infected with *Verticillium dahliae* strain Vd991, *GhCNGC1&18*-silenced plants remained an almost healthy status at 16 days post-inoculation (dpi), whereas mock control plants developed obvious yellowing leaves, representing a typical Verticillium wilt symptom (Figure 10D); by cutting the stems of these plants to examine the vascular wilt symptoms, it was found that the extent of vascular browning was much weaker in *GhCNGC1&18*-silenced plants than control plants (Figure 10E); moreover, the fungal biomass analysis of the stem tissues indicated that *GhCNGC1&18*-silenced plants developed significantly lower fungal biomass than mock control plants (Figure 10F). These results clearly showed that silencing *GhCNGC1&18* resulted in an enhanced resistance to fungal infection in Upland cotton. To further confirm *GhCNGC1&18*-mediated Verticillium wilt resistance, the activation of pathogenesis-related (PR) genes was investigated, which indicated that both the SA pathway marker gene *PR1* and the JA (jasmonic acid) pathway marker gene *PR3* were significantly upregulated in the *GhCNGC1&18*-silenced plants upon Vd991 inoculation (Figure 10G). It was noted that, under the mock control condition without infection, expression levels of both *PR1* and *PR3* genes in the *GhCNGC1&18*-silenced plants were significantly higher than those of the non-silenced control plants (Figure 10G), suggesting that the silencing of *GhCNGC1&18* caused the constitutive activation of *PR* genes, which are in agreement with those findings from mutants of orthologous CNGCs in *Arabidopsis* [48,68]. Altogether, the above results indicated that *GhCNGC1&18* are essential for growth and development, and they play negative roles during abiotic and biotic stress responses in Upland cotton.

**Figure 11:**
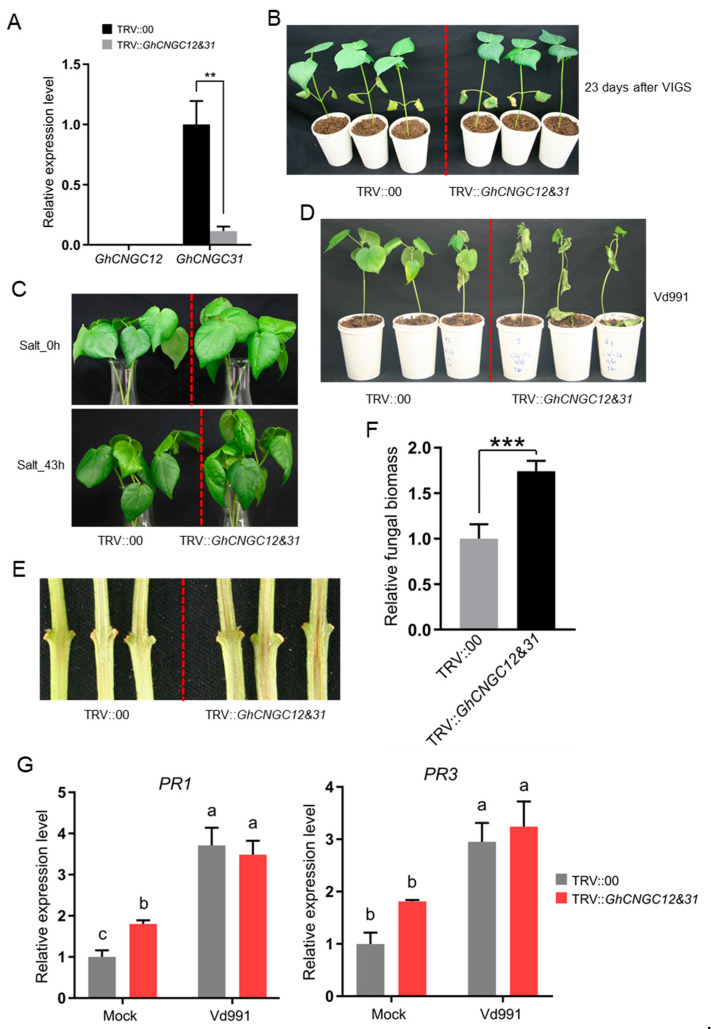
Effects of *GhCNGC12* and *GhCNGC31* simultaneous silencing in Upland cotton. Virus-induced gene silencing (VIGS) was performed by infiltration of cotyledons using *Agrobacterium* carrying the TRV::*GhCNGC12&31* vector that simultaneously targeted both *GhCNGC12* and *GhCNGC31* for silencing, or the empty vector (TRV::00) as mock control. (**A**) Relative expression levels of *GhCNGC12* and *GhCNGC31* in the silenced and control plants. *GhCNGC12* was not detectable. The second true-leaf samples were used for quantitative RT-PCR detection. (**B**) Phenotypes of *GhCNGC1&18*-silenced plants versus mock control plants. The silenced plants grow leaves with darken color and downward curled margin. (**C**) Salt stress resistance. *GhCNGC12&31*-silenced plants display obvious wilting symptoms as not yet seen with mock control plants after 43 h treatment of 200 mM NaCl. (**D**–**F**) Fungal disease resistance. In comparison with mock control plants, *GhCNGC12&31*-silenced plants develop more severe Verticillium wilt symptoms at 24 days post inoculation (dpi) with *V. dahliae* strain Vd991 (**D**), exacerbate vascular wilt symptoms of browning in the stem tissues at 24 dpi (**E**) and increase fungal biomass in the stem tissues at 24 dpi (**F**). (**G**) Expression of *PR* genes. The root samples for quantitative RT-PCR detection were collected from the Vd991-inoculated and mock control plants at 24 h post-inoculation. Data in (**A**,**F**,**G**) are mean ± SD (*n* = 3), Student’s *t*-test ** *p* < 0.01, *** *p* < 0.001; ANOVA followed by Tukey’s multiple comparisons test for (**G**), different letters indicate significant difference at *p* < 0.05.

Similarly, we characterized functions of *GhCNGC12&31* as the paired genes sharing the closest homology with *AtCNGC2* in Arabidopsis (Figure 1). Expression of *GhCNGC12* was not detectable in leaf samples, but *GhCNGC31* transcripts decreased by 89% more in *GhCNGC12&31*-silenced plants than in mock control plants (Figure 11A). Given that *GhCNGC12* was clearly expressed at varying levels during ovule/fiber development (Figure 7A), we cannot completely rule out the possibility that *GhCNGC12* may be expressed in other tissues (e.g., apical meristems) or induced upon stimulation, or even expressed at a barely detectable level by quantitative RT-PCR. *GhCNGC12&31*-silenced plants displayed few changes in phenotypes, except that the leaves appeared to have darker colors at an earlier growth stage (Figure 11B). Under the condition of salt stress, *GhCNGC12&31*-silenced plants started to exhibit obvious wilting symptoms after 43 h treatment, whereas mock control plants remained normal (Figure 11C), suggesting that *GhCNGC12&31* play positive roles during salt stress. Under the condition of Vd991 infection, *GhCNGC12&31*-silenced plants developed more severe Verticillium wilt symptoms (i.e., yellowing leaves, defoliation, and wilting) than mock control plants at 20 dpi (Figure 11D); the extent of vascular browning was much stronger in the stems of *GhCNGC12&31*-silenced plants than mock control plants at 24 dpi (Figure 11E); accordingly, fungal biomass in the stems of *GhCNGC12&31*-silenced plants was significantly higher than that in mock control plants (Figure 11F). These results supported that *GhCNGC12&31* positively contribute to Verticillium wilt disease resistance. A further detection of *PR* genes confirmed the significant elevation of both *PR1* and *PR3* genes in the *GhCNGC12&31*-silenced plants upon Vd991 inoculation; under mock control condition without infection, the *GhCNGC12&31*-silenced plants showed a significantly higher level of *PR1* expression compared to the non-silenced control plants, whereas *PR3* was increased without significant difference (Figure 11G). A comparison between *GhCNGC12&31*- and *GhCNGC1&18*-silenced plants indicated that Vd991 infection induced a similar level of both *PR1* and *PR3* expression in the *GhCNGC12&31*-silenced plants compared to that in the infected non-silenced control plants (Figure 11G); in contrast, Vd991 infection induced the highly significant elevation of both *PR1* and *PR3* expression in the *GhCNGC1&18*-silenced plants compared to that in the infected non-silenced control plants (Figure 10G), which may underline the different effects of pathogen resistance between *GhCNGC12&31*- and *GhCNGC1&18*-silenced plants.

In Arabidopsis, *AtCNGC2* and *AtCNGC4* are the only two members comprising the IV-B group of the CNGC family, and null mutants of both genes exhibited almost identical phenotypes, including severe dwarfing in stature, delayed flowering, loss of hypersensitive response (HR) cell death, and constitutive systemic resistance [48,68,90,91]; in addition, it was evident that both *AtCNGC2* and *AtCNGC4* may work in the same signaling pathway to regulate pathogen defense and floral transition [66]. While our above results demonstrated that *GhCNGC12&31* and *GhCNGC1&18* are required for growth and development, and they do cause similar leaf phenotypes, as well as the constitutive activation of *PR* genes by gene silencing; however, *GhCNGC12&31* and *GhCNGC1&18* played opposite roles during abiotic and biotic stress responses in Upland cotton. It was documented that the plant–pathogen interaction pathway played important roles in cotton defense response to *V. dahliae* infection, and that genes encoding the RLKs (receptor-like protein kinases) family members, including GhFLS2 (LRR receptor-like serine/threonine-protein kinase) and GhGsSRK (G-type lectin S-receptor-like serine/threonine-protein kinase), are highly up-regulated upon *V. dahliae* infection; however, VIGS experiments showed that *GhGsSRK*-silenced plants exhibited more severe symptoms compared with the vector control plants, whereas *GhFLS2*-silenced plants did not compromise cotton resistance to *V. dahlia* [92]. FLS2 has the most functional connection with GhCNGCs (Figure 6), which may be of significant importance to the coordinated regulation or complexity of pathogen responses in plants. Our results suggested that the closest orthologs of *CNGCs* in different plant species may play different roles in the specific genome background, which indicate the necessity of addressing the functions of *CNGCs* in a specific plant species for their potential application in breeding and improvement.

## 3. Materials and Methods

### 3.1. Plant Materials and Growth Conditions

Upland cotton cultivars “0–153” and “Han 8266” were used in the present study. Moreover, “0–153” is derived from introgressive hybridization between G. hirsutum (“Damian 2”) and G. arboreum (“Jinxian zhongmian”), and it has excellent fiber quality traits (an average fiber strength of 33.70 cN/tex, fiber length of 30.28 mm, and micronaire of 4.52). “Han 8266” is a commercial transgenic cultivar developed through the cross between a conventional variety “Han 4849” and a Bt (*Bacillus thuringiensis*) transgenic insect-resistant variety “Han 5158”, and it has high-yielding potential and wide adaptability, with resistance to insect and tolerance to Fusarium wilt (FW index 6.3~11.8) and Verticillium wilt (VW index 18.0~21.0). Cotton seeds were grown in potting soil (Pindstrup Mosebrug A/S, Ryomgård, Denmark) mixed with 20% (*v*/*v*) vermiculite, or grown hydroponically in Hoagland’s nutrient solution, at 25 °C, with 16 h light/8 h dark photoperiod in a growth room, except that the samples for cotton ovule culture were collected from “0–153” grown in an experimental field at Zhengzhou Research Base, State Key Laboratory of Cotton Biology, Zhengzhou, China.

### 3.2. Genome Data, Bioinformatics Identification and Protein Analysis

Genome sequence data for *Gossypium* ssp. were obtained from cotton databases (https://cottonfgd.org/; https://www.cottongen.org/ (accessed on 4 March 2020)), including *G. hirsutum* (NAU-NBI assembly v1.1 and annotation v1.1), *G. barbadense* (HAU assembly v2.0 and annotation v1.0), *G. arboreum* (CRI assembly v1.0 and annotation v1.0), *G. raimondii* (JGI assembly v2.0 and annotation v2.1), and *G. herbaceum* (WHU assembly v1.0 and annotation v1.0). To ensure the retrieved sequences of cotton CNGCs, when possible, cross-references between the genome sequences derived from different accessions of the same species were performed using the above cotton databases. *Arabidopsis thaliana* CNGCs were retrieved from the Arabidopsis Information Resources database (http://arabidopsis.org/ (accessed on 6 March 2020)). *Oryza sativa* CNGCs were retrieved from the rice genome database RAP-DB (https://rapdb.dna.affrc.go.jp/ (accessed on 6 March 2020)). The protein sequences of Arabidopsis CNGCs were used as queries for BLASTP search (*E*-value cutoff of 10^−10^) to identify potential CNGC genes in the cotton genomes. All retrieved non-redundant candidate genes were screened using the plant CNGC-specific motif: [LIMV0]-X(2)-[GSANCR]-X-[FVIYASCL]-X-G-X(0,1)-X(0,1)-[EDAQGH]-L-[LIVFA]-X-[WRCMLS0]-X-[LMSIQAFT0]-X(7,37)-[SAC]-X(9)-[VTIALMS]-X(0,1)-[EQDN]-[AGSVT]-[FYL]-X-[LIVF] [93]. Then, these genes were confirmed for containing CNBD (cyclic nucleotide-binding domain; pfam ID: PF00027) and TM/ITP (transmembrane or ion transport domains; pfam ID: PF00520) domains using the HMMER web server [94]. The conserved protein structures and/or domains of candidate CNGCs were further surveyed using InterProScan (http://www.ebi.ac.uk/interpro (accessed on 18 May 2020)) with the embedded signature databases (CDD, SMART, SUPERFAMILY, Pfam, CATH-Gene3D, PANTHER, PRINTS, PIRSF, TIGRFAM, PrositeProfiles, HAMAP, PrositePatterns, SFLD, SignalP, TMHMM, Phobius, Coils, and MObiDBLite). Potential genes encoding AKT/KAT-type potassium channels were excluded, which usually contain additional ankyrin repeats, except CNBD and TM/ITP domains [95,96].

All cotton CNGC genes were named according to their positions on the chromosomes in the genome. Protein isoelectric point (pI) and number of amino acids (aa) were calculated using ExPasy (https://web.expasy.org/ (accessed on 27 May 2020)). The protein subcellular location was predicted using WoLF PSORT (https://wolfpsort.hgc.jp/ (accessed on 28 May 2020)) [97].

### 3.3. Chromosomal Localization, Gene Duplication, and Phylogenetic Analysis

The chromosomal locations of cotton CNGC genes were determined according to the genome annotation data; the positions and relative distances on the chromosomes were visualized using TBtools software [98]. Synteny, collinearity, and gene duplication were analyzed using MCScanX (http://chibba.pgml.uga.edu/mcscan2/ (accessed on 6 June 2021)) [41]. Gene duplication was determined according to two criteria: (a) the length of the shorter aligned sequence covered > 70% of the longer sequence, and (b) the two aligned sequences shared > 70% amino acid sequence similarity [99]. Two genes separated by less than five intermediate genes in the 100 kb chromosomal fragment are considered to have undergone tandem duplication [100]. To detect the mode of selection forces acting on the protein, the ratio of the number of nonsynonymous substitutions per nonsynonymous site (Ka) to the number of synonymous substitutions per synonymous site (Ks) was calculated using TBtools with the Nei–Gojobori model [101]; generally, a Ka/Ks ratio > 1 indicates positive selection, the ratio < 1 implying negative or purifying selection, while the ratio = 1 indicates neutral evolution [43]. The density plots of Ks were analyzed and visualized using the lattice package in RStudio [102]. The estimated divergence time (T) of each duplicated gene pair was calculated as T = Ks/2r, in accordance with the neutral substitution rate of cotton (r = 2.6 × 10^−9^) [31]. The synteny relationship of orthologous CNGC genes in different species was constructed using the Dual Synteny Plotter (https://github.com/CJ-Chen/TBtools (accessed on 8 June 2021)) [103], and the results were visualized and optimized via RCircos [104]. The phylogenetic analysis was performed with the full-length amino acid sequences of CNGCs using MEGA X software (http://www.megasoftware.net/ (accessed on 4 July 2020)), wherein multiple sequence alignment was conducted using the ClustalW and the phylogenetic tree was generated using the neighbor-joining (NJ) method with 1000 bootstraps.

### 3.4. Gene Structure, Cis-Acting Regulatory DNA Elements and Protein Interaction Network

For the analysis of gene structure, intron/exon structure information was collected from the annotations of cotton genomes and analyzed using GSDS2.0 (http://gsds.cbi.pku.edu.cn/ (accessed on 2 September 2021)). To identify *cis*-acting regulatory DNA elements, the 1000 bp promoter sequence upstream from translation start site of the CNGC gene was analyzed using the PLACE database (http://dna.affrc.go.jp/PLACE/?action=newplace/ (accessed on 7 September 2021)). To construct a functional protein association network, protein–protein interactions were determined using STRING (v11.0; https://string-db.org/cgi/input.pl (accessed on 19 September 2021)) with the highest confidence score >0.9, and the results were depicted by a network using Cytoscape (v3.8.2; https://cytoscape.org/ (accessed on 20 September 2021)).

### 3.5. Plant Treatments with Hormones and Stress Conditions

For hormonal treatment, 3-week-old seedlings (*G. hirsutum* “0–153”) were sprayed with a sterile distilled water solution containing salicylic acid (SA), methyl jasmonate (MeJA), 1-aminocyclopropane-1-carboxylic acid (ACC), or abscisic acid (ABA) at the indicated concentration, and then immediately covered overnight using a transparent dome; the above-ground tissue samples were collected 24 h after spraying, along with the mock control. For fungal infection, the stock of *Verticillium dahliae* stain Vd991 was first activated by growth on PDA (potato dextrose agar) for 5–7 days at 25 °C under the dark condition; then, the mycelia were collected and cultured in liquid Czapek’s medium with shaking (150 rpm); finally, conidia were harvested by centrifugation, washed with sterile water, and adjusted to a final concentration of 1 × 10^7^ conidia/mL sterile water using a hemocytometer. Three-week-old seedlings (*G. hirsutum* “Han 8266”) grown in a 9-ounce paper cup were inoculated by root dipping in 30 mL conidial suspension per seedling plant, and whole plant samples were collected 3 days post-inoculation (dpi). For salt stress treatment, cotton seeds (*G. hirsutum* “0–153”) were germinated for 3 days, and then grown hydroponically in Hoagland’s nutrient solution containing 200 mM NaCl for 2 weeks before the collection of whole plant samples.

### 3.6. VIGS Analysis and Detection of Stress Resistance

An *agrobacterium*-mediated virus-induced gene silencing (VIGS) assay was performed using the tobacco rattle virus (TRV) system [89,105]. Specifically, VIGS target fragments of *GhCNGCs* were determined to ensure the specificity by BLAST search against *G. hirsutum* genome sequence, and approximately 400–500 bp fragments were amplified from *G. hirsutum* genomic DNA using gene-specific primers (Supplementary Table S1). Each of the fragments was cloned into vector pTRV2, generating pTRV2 derivatives (pTRV2::*GhCNGCs*), which were subsequently introduced into *Agrobacterium tumefaciens* GV3101. For achieving a high silencing efficiency, two target fragments from different regions of each gene were separately cloned to generate the derived *Agrobacterium* cultures, which were used by a mixture of equal proportions. *Agrobacterium* was cultured in LB medium containing 50 mg/L kanamycin and 20 mg/L rifampicin overnight at 28 °C in a shaker (200 rpm), and then sub-cultured in the same fresh medium overnight (OD_600_ = 0.8~1.2). Cells were pelleted by centrifugation at 4000× *g* for 10 min, resuspended with OD_600_ of 1.0 in infiltration buffer containing 10 mM MgCl_2_, 10 mM 2-(N-morpholino)-ethanesulfonic acid (MES), and 200 µM acetosyringone. Cell suspensions of *Agrobacterium* carrying pTRV2 or pTRV2 derivatives were mixed with pTRV1 in a 1:1 ratio, and incubated at room temperature for at least 3 h. Cotton seedlings with fully expanded cotyledons were used to infiltrate the cotyledons with the mixed culture using a 1 mL needleless syringe. Immediately after infiltration, plants were watered, covered with a plastic dome, and shaded from light using a piece of black plastic cloth overnight. The effectiveness of the VIGS assay was evaluated by silencing the phytoene desaturase gene (*PDS*) as a positive control, resulting in visible leaf photo-bleaching [106]. When *PDS*-silenced plants displayed white leaves at approximately 2 weeks post-infiltration, experiments for the evaluation of stress resistance were conducted. The second true leaf samples were collected for RNA extraction and interference efficiency detection by quantitative RT-PCR through the comparison of gene expression levels in the silenced plants and mock control plants. All the experiments were conducted at least three times independently.

For the evaluation of salt stress resistance, VIGS-treated plants at the three leaves stage were transplanted with roots soaking in a water solution containing 200 mM NaCl, as reported previously [107]. For examining fungal disease resistance, VIGS-treated plants, for about two to three weeks (i.e., after two true leaves fully-expanded), were inoculated with Vd991 as described above, and *Verticillium* wilt symptoms were investigated; seedling shoots were cut to investigate vascular wilt symptoms under a microscope [108]; relative fungal biomass in the stem tissues was quantified by quantitative PCR using primers specific to the internal transcribed spacer region (ITS) of 5.8S ribosomal RNA gene in *V. dahliae* and *GhUBQ7* (GenBank: DQ116441) gene in *G. hirsutum* for sample equilibration (Supplementary Table S1), as we have described previously [7].

### 3.7. Quantitative RT-PCR

Total RNA was isolated using the EASYspin Plus Plant RNA Kit (Aidlab, Beijing, China) following manufacturer’s instructions. Reverse transcription (RT) was performed using the HIScript^®^ III RT SuperMix for qPCR (+gDNA wiper) Reagent Kit (Vazyme Biotech, Nanjing, China). A quantitative PCR analysis was carried out using the ChamQ^TM^ Universal SYBR^®^ qPCR Master Mix (Vazyme Biotech) and gene-specific primers (Supplementary Table S1) in a LightCycler^®^ 480II PCR system (Roche, Basel, Switzerland). The cycling conditions were 30 s at 95 °C, 40 cycles of 10 s at 95 °C, and 30 s at 60 °C. The specificity of amplified products was monitored by melting curve analysis, and verified by agarose gel electrophoresis. Relative expression levels of genes were normalized to *GhUBQ7* as an internal control and calculated using the 2^−∆∆Cq^ method. When necessary, the house-keeping gene *GhACT7* was used for plate-to-plate equilibration.

### 3.8. Ovule Culture, Drug Treatments and Transcriptomic Profiling

The chemicals forskolin (FSK), 8-Br-cAMP, and 2′,3′-dideoxyadenosine (DDA) were purchased from Sigma-Aldrich, Shanghai, China. Then, 8-Br-cAMP was solubilized as a 50 mM stock in water and filter sterilized; FSK and DDA were prepared as 50 mM and 200 mM stock in DMSO, respectively. The stock solutions were aliquoted and stored at −80 °C.

Cotton ovules culture was conducted as previously described with minor modification [109]. Briefly, the bolls at the stages of 0 or 1 day post anthesis (DPA) were collected with anthocaulus from cotton plants (*G. hirsutum* “0-153”); ovaries were surface-sterilized and dissected under sterile conditions; intact ovules were immediately placed into liquid Beasley and Ting (BT) medium containing 18 g/L glucose, 3.6 g/L fructose, 5 μM indole-3-acetic acid (IAA) and 0.5 μM GA_3_, and then incubated at 32 °C in the dark without agitation. The fiber should be easily visible after 4~5 days of culture. For drug treatments, DDA (50 or 100 µM), FSK (10 or 50 µM), or 8-Br-cAMP (10 or 50 µM) were added with the indicated final concentrations in the medium. After 6 days of culture, the ovules were harvested for RNA extraction. Three biological replicates were performed.

Total RNAs were isolated using the EASYspin Plus Plant RNA Kit, as described above. Preparation of sequencing libraries, instrumental platform, raw reads cleaning, transcripts assembly and alignment, annotation and quantification were all performed as we previously described [7], except that the reference genome of *G. hirsutum* ‘TM-1’ (NAU-NBI_v1.1_a1.1; https://www.cottongen.org/ (accessed on 20 September 2020)) was used. FPKM (fragments per kilobase of exon model per million mapped fragments) for each gene model were calculated for an estimate of expression level. Differential gene expression was determined with an absolute value of log2 (fold change) >1 and a false discovery rate (FDR) adjusted *p*-value < 0.05.

RNA sequencing data of *G. hirsutum* ‘TM-1’ during growth and development were downloaded from the NCBI Sequence Read Archive under accession number PRJNA490626 [31], and used for analyzing gene expression patterns.

### 3.9. Statistical Analysis

Data for quantification analyses were presented as mean ± standard deviation (SD) with three biological replicates. Student’s *t* test or analysis of variance (ANOVA) followed by Tukey’s multiple comparisons test were performed to determine significant differences between the means using GraphPad Prism 8.0.2 (https://www.graphpad.com/ (accessed on 14 October 2021)).

## 4. Conclusions

In the present study, genome-wide analyses have identified a total of 40, 41, 20, 20, and 20 CNGC genes in *G. hirsutum*, *G. barbadense*, *G. herbaceum*, *G. arboreum*, and *G. raimondii*, respectively. These genes are highly conserved during evolution across all the five cotton species above, and they are classified into five phylogenetic groups (I, II, III, IV-A and IV-B), conforming to the CNGC family in other plant species. Most members of the CNGC family in cotton are localized to plasma membrane, except some of them residing in the nucleus, mitochondria, and chloroplasts; however, a considerable portion (8/20) of the CNGC family in *G. herbaceum* may target chloroplasts. The CNGC genes are distributed on most chromosomes in the cotton genome, with a highly similar pattern across different species, and the family expansion is mainly derived from segmental duplication under purifying selection, and show very similar patterns of syntenic relationships between the species. Generally, the most closely related CNGC family members in the phylogenetic tree tend to have a more similar gene structure, while the CNGC family in *G. hirsutum* and *G. herbaceum* seems to display greater gene structure variations among the five cotton species. Further analyses of the CNGC family genes in *G. hirsutum* confirmed that they are extensively expressed in various tissues, and at different developmental stages. Each *GhCNGC* gene contains a variety of *cis*-acting elements residing in the promoter regions, which are implicated in response to phytohormones, biotic and abiotic stimuli; however, the CNGC family genes each showed distinct patterns, per the composition and copy number of *cis*-acting elements. Accordingly, quantitative RT-PCR detection unveiled diverse and altered expression patterns of *GhCNGCs* upon treatments of hormones (ABA, SA, MeJA and ethylene), salt stress, and *V. dahlia* infection in cotton plants; additionally, a set of *GhCNGCs* were identified in response to cAMP signaling during cotton fiber development. A functional protein association network of the CNGC Family in *G. hirsutum* was established, demonstrating the linkages of GhCNGCs with a few crucial proteins in plant immune responses (FLS2, RSTK and MOL), growth and development (CLV2 and TAD3), as well as with various transporter and exchanger proteins implicated in response to genetic, hormonal, and environmental cues, in addition to the dynamic interactions between different GhCNGCs. The silencing of both the homoeologous gene pair *GhCNGC1&18* and *GhCNGC12&31* impaired plant growth and development; however, *GhCNGC1&18*-silenced plants enhanced Verticillium wilt resistance and salt tolerance, whereas *GhCNGC12&31*-silenced plants showed the opposite effects. Collectively, these findings enrich our knowledge on the CNGCs family and its association with cAMP signaling, about which almost nothing is known currently in cotton, and thus pave the foundation for elaborating the biological functions and utilization of CNGCs in cotton breeding and genetic improvement.

## Figures and Tables

**Table 1:** Effects of modulating cAMP signaling on *GhCNGCs* expression during cotton fiber development.

Gene Name	8-Br-cAMP				Forskolin			DDA		
	CK	10 µM	50 µM		CK	10 µM	50 µM	CK	50 µM	100 µM
*GhCNGC1*	62.22	65.20	61.87		56.03	61.83	74.25	115.83	44.41▼	22.85▼↓
*GhCNGC3*	0.42	0.33	0.55		0.38	0.51	0.29	0.70	0.33▼	0.44
*GhCNGC5*	1.06	1.03	0.84		0.72	1.45	1.56▲	1.44	1.71	3.97▲↑
*GhCNGC11*	0.08	0.04	0.07		0.00	0.15	0.29	0.54	0.16▼	0.18
*GhCNGC13*	63.22	62.65	65.23		55.47	70.20	74.87	91.73	52.14	30.12▼
*GhCNGC15*	3.05	2.73	3.13		3.31	3.49	4.15	9.36	5.00	4.05▼
*GhCNGC16*	0.50	0.43	0.35		0.61	0.58	0.75	0.70	0.54	1.31 ↑
*GhCNGC18*	21.37	21.95	21.35		20.39	24.12	39.67	49.06	14.39▼	6.73▼↓
*GhCNGC20*	1.90	1.95	1.75		1.88	1.83	1.19	2.05	0.89▼	0.63▼
*GhCNGC26*	0.53	0.33	0.43		0.28	0.53	0.63▲	0.43	0.52	0.80
*GhCNGC32*	92.51	88.37	97.02		75.75	103.16	99.36	117.54	94.00	57.00▼
*GhCNGC35*	0.81	0.92	0.89		0.95	0.93	1.51	4.15	1.36▼	1.34▼
*GhCNGC39*	7.33	7.98	7.07		8.18	6.48	5.62	3.80	1.61▼	1.65▼

Note: data are from cotton ovule culture experiments under treatments of forskolin, 2′,3′-dideoxyadenosine (DDA) or 8-Br-cAMP, showing the estimates of gene expression levels by FPKM (fragments per kilobase of exon model per million mapped fragments) from transcriptome sequencing. Experiments with 8-Br-cAMP and forskolin treatments were conducted in parallel, whereas experiment with DDA treatment was completed in a different year. ▲ and ▼ indicate significant up- and down-regulation in comparison with the mock control (CK), respectively; **↑** and **↓** indicate significant up- and down-regulation compared to the treatments of lower concentrations, respectively.

## Data Availability

The data and materials that support the findings of this study are available from the corresponding author upon reasonable request.

## Supplementary Materials

The following supporting information can be downloaded at: https://www.mdpi.com/article/10.3390/ijms23042041/s1.
