# Supplementary material for: Dynamic Expression, Differential Regulation and Functional Diversity of the CNGC Family Genes in Cotton
Source: Int J Mol Sci. 2022 Feb 12;23(4):2041. doi: 10.3390/ijms23042041 (PMC8878070; doi:10.3390/ijms23042041)
Supplement: Supplementary file 1 [file ijms-23-02041-s001.zip › Figure S1-S4 Phylogenetic relationships of the CNGC family members in cotton.pdf]

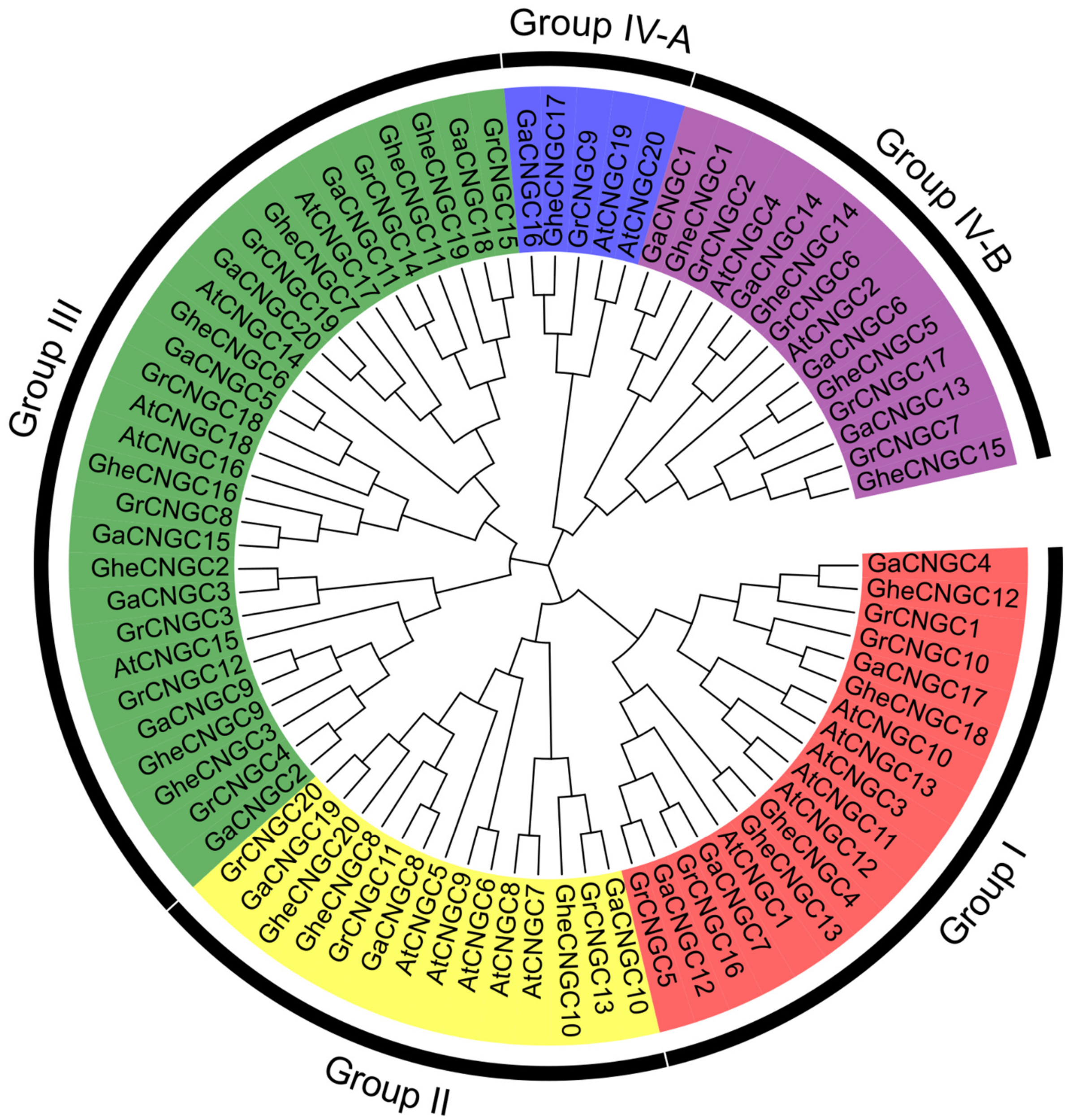

Figure S1. Phylogenetic relationships of the CNGC family members in the diploid species of cotton. The amino acid sequences of cyclic nucleotide-gated channels (CNGCs) were aligned with ClustalW, and a consensus tree was generated by the Neighbor-Joining (NJ) method with 1000 bootstraps using MEGA X software (Kumar et al. *Mol Biol Evol*. 2018, 35:1547-1549). The cotton CNGCs were clustered into four groups (I, II, III, IV) and two subgroups (IV-A and IV-B), conforming to the classification of CNGCs in *Arabidopsis thaliana* (Talke et al. *Trends Plant Sci*. 2003, 8:286-293). *G. herbaceum*, GheCNGC1-20; *G. arboreum*, GaCNGC1-20; *G. raimondii*, GrCNGC1-20; *A. thaliana*, AtCNGC1-20.

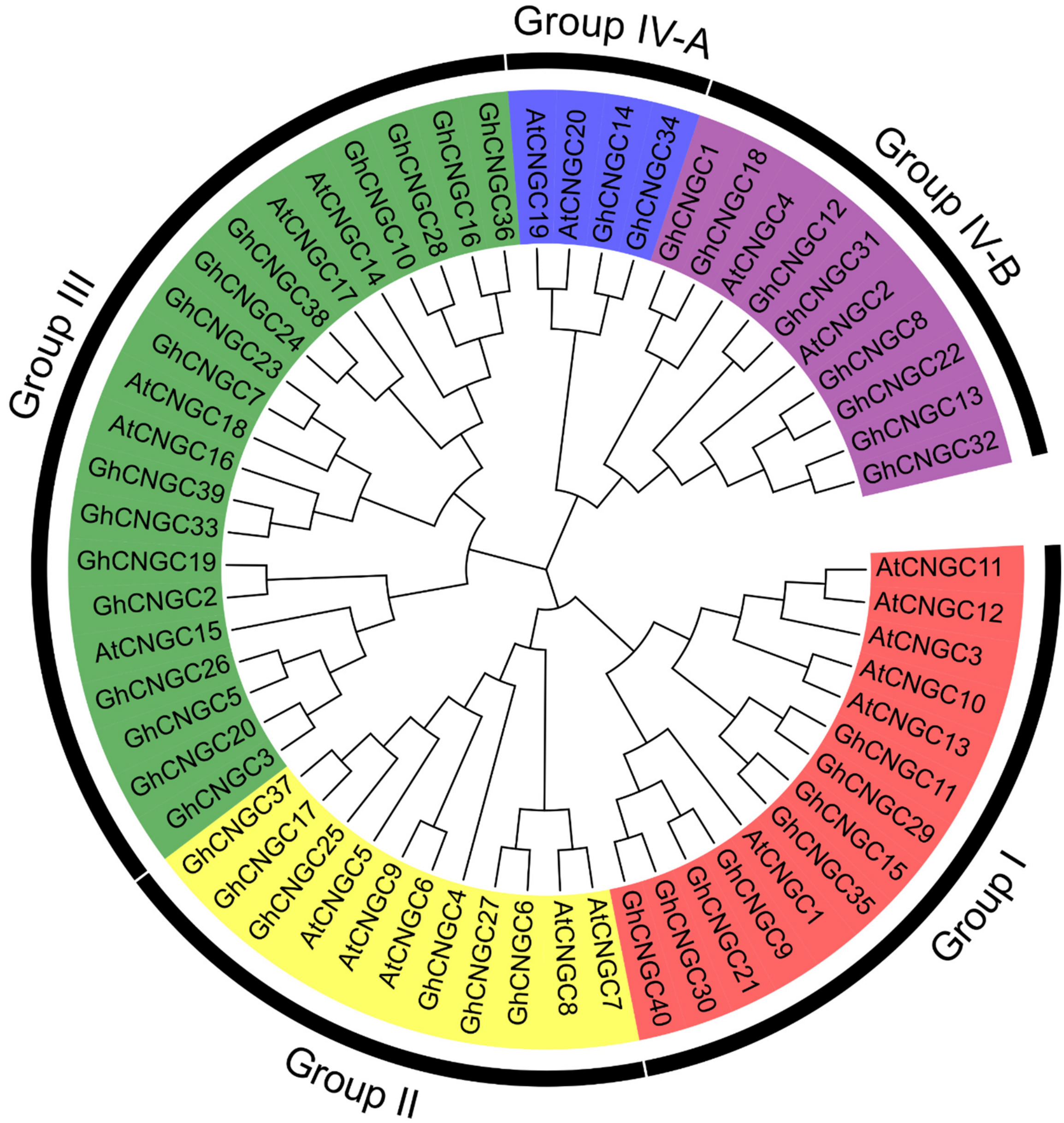

Figure S2. Phylogenetic relationships of the CNGC family members in the tetraploid species *Gossypium hirsutum*. The amino acid sequences of cyclic nucleotide-gated channels (CNGCs) were aligned with ClustalW, and a consensus tree was generated by the Neighbor-Joining (NJ) method with 1000 bootstraps using MEGA X software (Kumar et al. *Mol Biol Evol*. 2018, 35:1547-1549). The CNGCs in *G. hirsutum* were clustered into four groups (I, II, III, IV) and two subgroups (IV-A and IV-B), conforming to the classification of CNGCs in *Arabidopsis thaliana* (Talke et al. *Trends Plant Sci*. 2003, 8:286-293). *G. hirsutum*, GhCNGC1-40; *A. thaliana*, AtCNGC1-20.

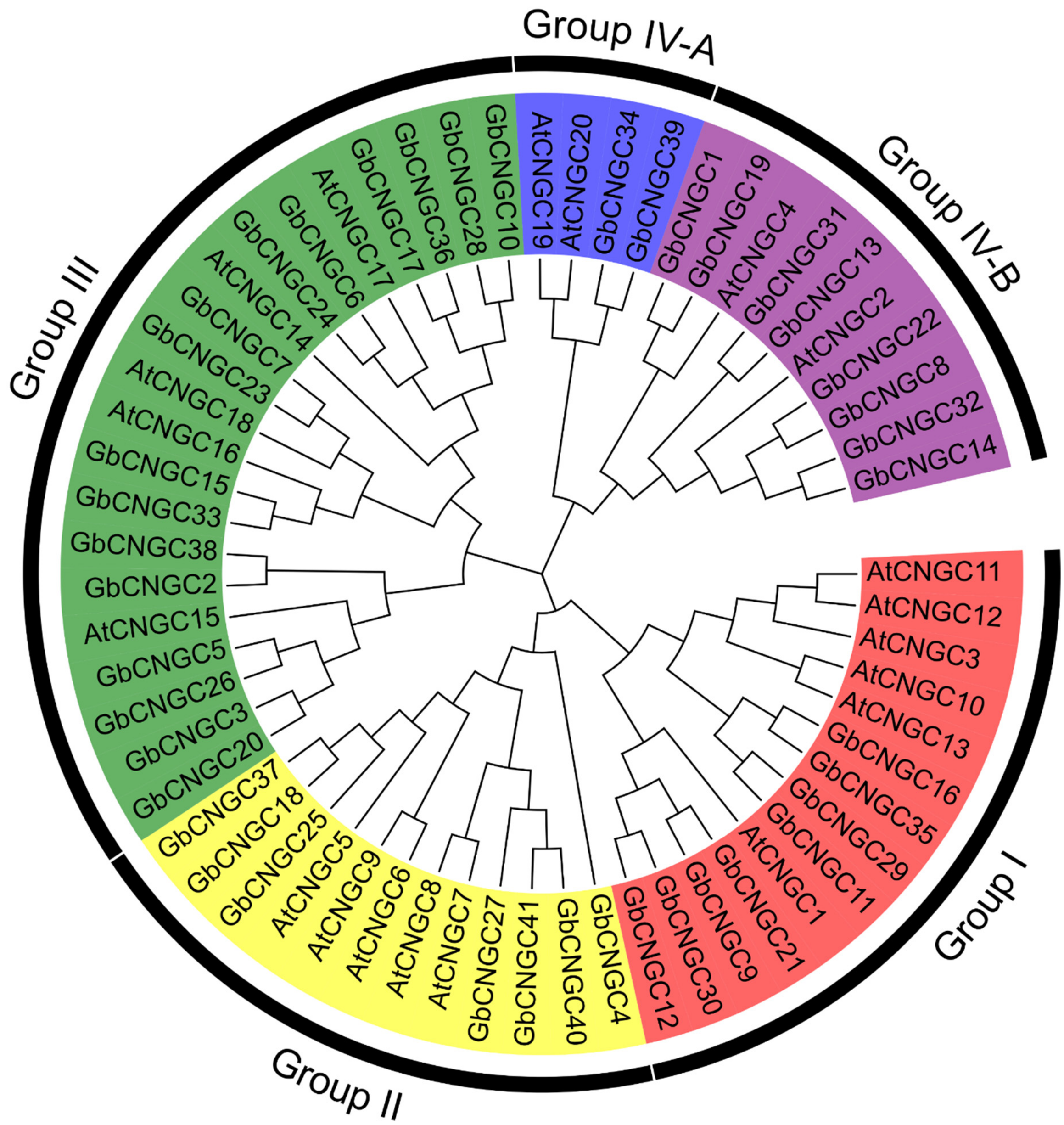

Figure S3. Phylogenetic relationships of the CNGC family members in the tetraploid species *Gossypium barbadense*. The amino acid sequences of cyclic nucleotide-gated channels (CNGCs) were aligned with ClustalW, and a consensus tree was generated by the Neighbor-Joining (NJ) method with 1000 bootstraps using MEGA X software (Kumar et al. *Mol Biol Evol*. 2018, 35:1547-1549). The CNGCs in *G. barbadense* were clustered into four groups (I, II, III, IV) and two subgroups (IV-A and IV-B), conforming to the classification of CNGCs in *Arabidopsis thaliana* (Talke et al. *Trends Plant Sci*. 2003, 8:286-293). *G. barbadense*, GbCNGC1-41; *A. thaliana*, AtCNGC1-20.

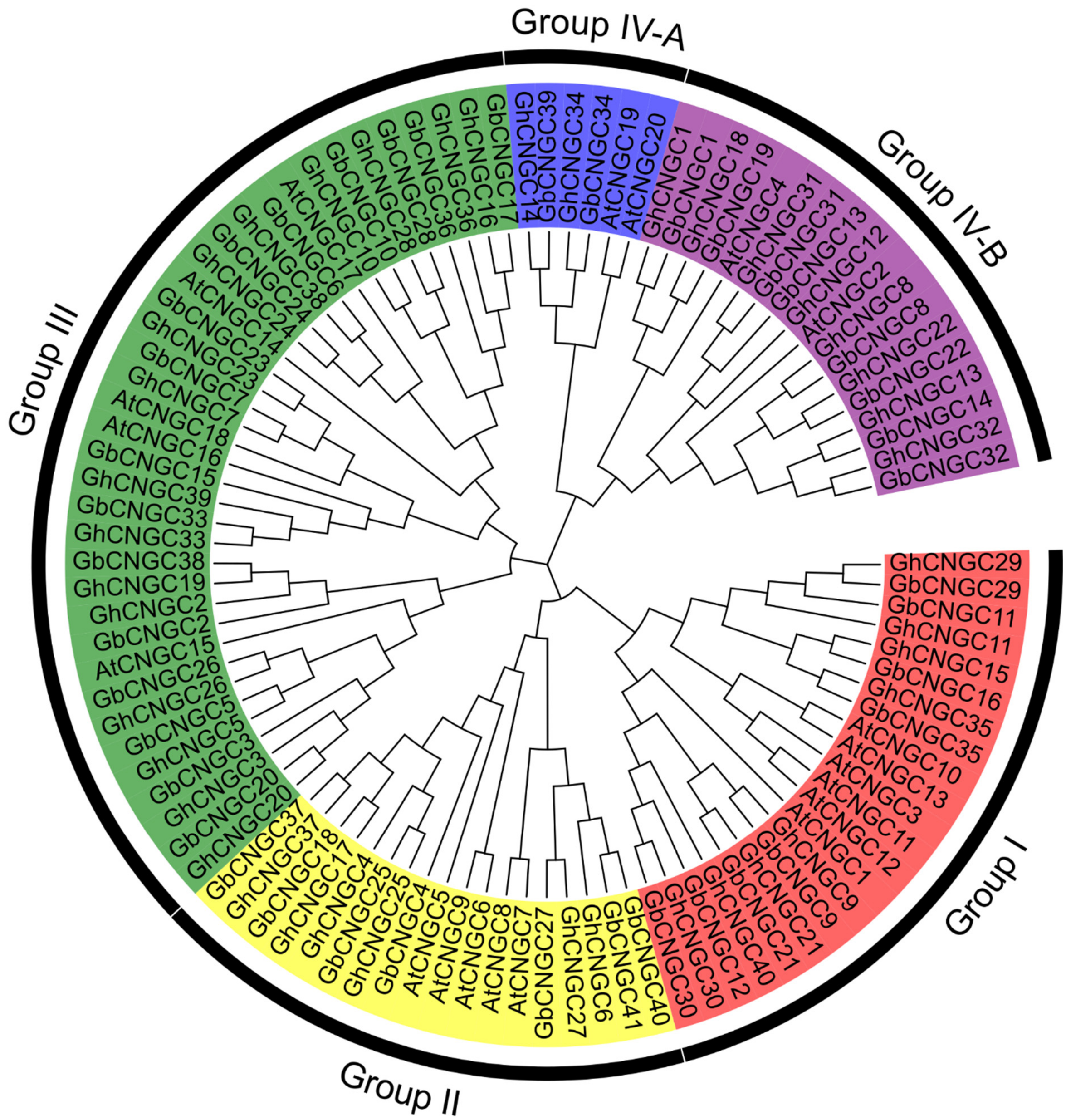

Figure S4. Phylogenetic relationships of the CNGC family members in the tetraploid species of cotton. The amino acid sequences of cyclic nucleotide-gated channels (CNGCs) were aligned with ClustalW, and a consensus tree was generated by the Neighbor-Joining (NJ) method with 1000 bootstraps using MEGA X software (Kumar et al. *Mol Biol Evol*. 2018, 35:1547-1549). The cotton CNGCs were clustered into four groups (I, II, III, IV) and two subgroups (IV-A and IV-B), conforming to the classification of CNGCs in *Arabidopsis thaliana* (Talke et al. *Trends Plant Sci*. 2003, 8:286-293). *G. hirsutum*, GhCNGC1-40; *G. barbadense*, GbCNGC1-41; *A. thaliana*, AtCNGC1-20.
