# Supplementary material for: Dynamic Expression, Differential Regulation and Functional Diversity of the CNGC Family Genes in Cotton
Source: Int J Mol Sci. 2022 Feb 12;23(4):2041. doi: 10.3390/ijms23042041 (PMC8878070; doi:10.3390/ijms23042041)
Supplement: Supplementary file 1 [file ijms-23-02041-s001.zip › Table S1 Primers used in this study.pdf]

Table S1. Primers used in this study.

Gene Name	Sequence (5'→3')	Size (bp)
GhCNGC1	Forward: TTATTGCAATAATGATGAATTAGGTTC	112
	Reverse: ATGAGAGAGTGTCGAATATAAACATAC	
GhCNGC2	Forward: AGTTTTGGTGATTACTAGTGTAGTT	83
	Reverse: ATACTCCTGGTTTGAGTTTATCTG	
GhCNGC3	Forward: TAATCAATGGAGTCAAGTAAGACTAAG	112
	Reverse: TCGGTTCATCACGATTTTTAATTCG	
GhCNGC4	Forward: CTGAATCCACTCCTGAAAAGAATCC	138
	Reverse: TGTCTCTACAAGTTGCAGTGTGATG	
GhCNGC5	Forward: AGTTAAGTCCTCGTAAGTTACGAGT	136
	Reverse: GTATGCATTCTTGGTTGAACTCTCA	
GhCNGC6	Forward: CGGTTTTGATTGGTGTGCGCTG	99
	Reverse: GGCCGGTGCAATCGATACGTAC	
GhCNGC7	Forward: AGTTGAGTGATTGAAATGAAACCAATAT	139
	Reverse: ATGTTCAGATCCATCCATCCAATTTATT	
GhCNGC8	Forward: TGAAATGCAATCCACCATACAGGA	124
	Reverse: AACGTGGAAATCATAGGACATAGT	
GhCNGC9	Forward: CTGTCAAATAAGTAGTCCGCATAAC	141
	Reverse: CAATGGGGTAACAAGATAATCTAAG	
GhCNGC10	Forward: AATCTTGGACAGCTGGTACACC	171
	Reverse: ATATTGGTACACCGGTGATTACG	
GhCNGC11	Forward: TGCATAAAGAGTGGCTCCGAGAC	101
	Reverse: TTCGTGAAGCAGAGGATAAGCTG	
GhCNGC12	Forward: ACTCCTCCATTCGAGACAGTGA	152
	Reverse: GCATGGACCGTGACAAATATAC	
GhCNGC13	Forward: AGTACGCACAAGACCATTCATCAAA	143
	Reverse: ATCGACCGAGACGAATAAAGAATAG	
GhCNGC14	Forward: CTCAATCAATGGCGTATTTCATATCA	194
	Reverse: TGTCATACACTGCGAGAAATAACTCA	
GhCNGC15	Forward: CAAATCAACTTATGGGGTCAATTATG	108
	Reverse: GAGATTTTTACATTTCGGATAATTCAA	
GhCNGC16	Forward: TCAGAATATTCACCGCCCTTATC	152
	Reverse: AACTCAGAGGTTTACAAGTCAAGA	
GhCNGC17	Forward: GAAAACGATTGATTCATGTTAGTATCA	188
	Reverse: TACATTGTAGCAAGAGTTCGATTAC	
GhCNGC18	Forward: CATATCCGACACTCTCTCGTATCTG	188
	Reverse: CCACTTTTCTGCTTTAGAGACACTC	
GhCNGC19	Forward: ATGAAAAGAAAAAATGTATGGTGACTA	112
	Reverse: TTAGACTCCTGGTAAAGCAACACT	
GhCNGC20	Forward: TTTGGATAAAGGAGAAACTTAAGTATA	145
	Reverse: GTGTAGGTATTATTAGTCAGTGAACAA	
GhCNGC21	Forward: ATGCAGGAGCAGCCGAAGTCA	123
	Reverse: AGGGTTCATAAATGCGCCAACA	
GhCNGC22	Forward: ATGGGACATGGTACTCCACTAA	105
	Reverse: CGGAATCCACCGTTAAGTACAA	
GhCNGC23	Forward: ATAAGATGAAATACAAGTAAAGCCATA	162

	Reverse: AGTTGAGTGAGTGTTGATGTAATTTA	
GhCNGC24	Forward: CTATCGGTTAGGAAGAAAGACGA	67
	Reverse: TGGGCTGCTTTGTTTGTAGTCAA	
GhCNGC25	Forward: TCTCTACAAGTTGCAGTGTGATGA	138
	Reverse: CTGAAC TCACTCCTGAAATTTACA	
GhCNGC26	Forward: AATATATCATTTCCAGTTAAGTCCTA	151
	Reverse: AATTCAAGTAATCAACATCCTTCCAT	
GhCNGC27	Forward: TGAAATCATCGTGGTTGGTTAATCA	159
	Reverse: GGTGCACCATGTTCCGTACGA	
GhCNGC28	Forward: GATTCAATAATGTTAAAAAGTGTGACA	169
	Reverse: TATATATGGGCACTAAAGAACGGCA	
GhCNGC29	Forward: TTCCGCCAATTACCAGCGACA	145
	Reverse: AAGTCAAGGGAAGAGCCTGCA	
GhCNGC30	Forward: ATCGTACTTGACACTAATATCAACA	184
	Reverse: TTCCTGACAATTTAGTGCTCCTTA	
GhCNGC31	Forward: TTACTCCACCTCAACCTGATTCA	146
	Reverse: GTGCATGGACCGTGACAGATATA	
GhCNGC32	Forward: GCTGTGCTAAACACGAGTATATGT	142
	Reverse: CTACGATGACCTCAAGTCTAAGAA	
GhCNGC33	Forward: AGAATATAAAGCCCTTGCCAACGCA	71
	Reverse: GTTTGGAGAACTTGCAACTCAGATA	
GhCNGC34	Forward: ACCTGGTTAAAGCTGAATTGGGTA	142
	Reverse: TTTGAGTCATTGCTTCTTCTTCTTA	
GhCNGC35	Forward: TATTGCAACTCGCTTGAAAGAAC	134
	Reverse: CAGCTACTGTTTGAGTCAGGA	
GhCNGC36	Forward: TCAATAACAATGGCAGTAGTACAGA	118
	Reverse: GGAATTCTACTATTACTATTACTATCA	
GhCNGC37	Forward: CAGTTTGGAAATACAGTTGTAAGTA	80
	Reverse: TAGGCATAAGAATTAATGAAGCAAC	
GhCNGC38	Forward: GTGGAACCAAATGATATGACACCA	143
	Reverse: ACCAGTCTGACAACCATCCTGTTA	
GhCNGC39	Forward: CTCAAACATAATCACATAGGATCGA	120
	Reverse: AAAGAAAACTGTAGCATGCTTATAG	
GhCNGC40	Forward: CATCATATACAGACCTCATACTCA	137
	Reverse: GCAGGTAGGTGAGAGACTTA	
GhACT7	Forward: GATTCCGGTGACGGTGTTTC	152
	Reverse: GCAGTGGTGGTGAACATGTAAC	
GhUBQ7	Forward: CGAGTCTTCGGACACCATTGA	258
	Reverse: GGACAGCGAGCTTGACCTTC	
GhCNGC1&18 (1)	Forward: ATCCTTCACGTGCAACGCCCATGCA	439
	Reverse: CTCTTGATCATTTTGAGCTGGAGGCAC	
GhCNGC1&18 (2)	Forward: GACCCAGTACAAAGAATGCTAT	400
	Reverse: TCCGAGGCCTAATGAATGACA	
GhCNGC12&31 (1)	Forward: TGAGAGAGGAACAATACAAGCTA	473
	Reverse: GAGAATCTTAACAGCGAGAGAGT	
GhCNGC12&31 (2)	Forward: AAGGAGATCCGGTGCTACGA	425
	Reverse: TAAGTCGATCTTCACTTCCACCA	
GhPR1	Forward: AAGAATGTGGGTTAGTGAGAGGGT	90

	Reverse: ACCACTTGAGTATAATGCCCGC	
GhPR3	Forward: GATGACTCCACAATCACCGAAGC	172
	Reverse: GCGGTCTTCTACCTGGGCATT	
Vd_ITS	Forward: CGGTGTTGGGGATCTACGTC	129
	Reverse: CGGGCTTGTAGGGGGTTTAG	
