# Supplementary material for: Dynamic Expression, Differential Regulation and Functional Diversity of the CNGC Family Genes in Cotton
Source: Int J Mol Sci. 2022 Feb 12;23(4):2041. doi: 10.3390/ijms23042041 (PMC8878070; doi:10.3390/ijms23042041)
Supplement: Supplementary file 1 [file ijms-23-02041-s001.zip › Table S2-S6 List of the CNGC family genes in different cotton species.pdf]

**Table S2.** List of the CNGC family genes in *Gossypium hirsutum.*

| Gene Name | Gene ID | Chr:Location(strand) | AA | pI | D&M | Group | Subcellular Localization |
|---|---|---|---|---|---|---|---|
| *GhCNGC1* | Gh_A01G0520 | A01:8277150..8284315(+) | 709 | 9.02 | a,b,c,d | IV-B | Plasma membrane |
| *GhCNGC2* | Gh_A01G1841 | A01:98325294..98327741(+) | 701 | 8.99 | a,b,c,d | III | Plasma membrane |
| *GhCNGC3* | Gh_A03G0266 | A03:4244529..4247150(+) | 703 | 9.10 | a,b,c,d | III | Plasma membrane |
| *GhCNGC4* | Gh_A05G0448 | A05:4940335..4941778(-) | 313 | 9.17 | b,d,e | II | Nuclear |
| *GhCNGC5* | Gh_A05G0903 | A05:8902041..8905061(-) | 713 | 9.21 | a,b,c,d | III | Plasma membrane |
| *GhCNGC6* | Gh_A05G1012 | A05:10147786..10152216(+) | 748 | 8.95 | a,b,c,d,e | II | Plasma membrane |
| *GhCNGC7* | Gh_A05G3130 | A05:80780849..80783618(-) | 736 | 8.37 | a,b,c,d | III | Plasma membrane |
| *GhCNGC8* | Gh_A05G3196 | A05:83677517..83680754(-) | 695 | 9.33 | a,b,c,d | IV-B | Plasma membrane |
| *GhCNGC9* | Gh_A05G3588 | A05:91763003..91768450(+) | 695 | 9.30 | a,b,c,d,e | I | Plasma membrane |
| *GhCNGC10* | Gh_NAU_A06* | A06:41590465..41594219(-) | 709 | 9.11 | a,b,c,d | III | Plasma membrane |
| *GhCNGC11* | Gh_A07G1267 | A07:29283930..29286198(-) | 599 | 9.23 | a,b,c,d | I | Plasma membrane |
| *GhCNGC12* | Gh_A09G0110 | A09:2549787..2554380(-) | 524 | 9.35 | a,b,d | IV-B | Plasma membrane |
| *GhCNGC13* | Gh_A09G0111 | A09:2575761..2581410(+) | 766 | 9.57 | a,b,c,d | IV-B | Plasma membrane |
| *GhCNGC14* | Gh_A11G0014 | A11:194792..217283(-) | 786 | 8.84 | a,b,c,d | IV-A | Plasma membrane |
| *GhCNGC15* | Gh_A12G1004 | A12:61310236..61315344(+) | 708 | 9.59 | a,b,c,d,e | I | Plasma membrane |
| *GhCNGC16* | Gh_A13G1579 | A13:74640085..74642700(+) | 703 | 8.95 | a,b,c,d | III | Plasma membrane |
| *GhCNGC17* | Gh_A13G1614 | A13:74974260..74978836(+) | 743 | 9.02 | a,b,c,d,e | II | Plasma membrane |
| *GhCNGC18* | Gh_D01G0533 | D01:6643589..6650779(+) | 709 | 9.02 | a,b,c,d | IV-B | Plasma membrane |
| *GhCNGC19* | Gh_D01G2082 | D01:59853608..59856045(+) | 726 | 8.92 | a,b,c,d | III | Plasma membrane |
| *GhCNGC20* | Gh_D03G1301 | D03:41182870..41185544(-) | 721 | 9.01 | a,b,c,d | III | Plasma membrane |
| *GhCNGC21* | Gh_D04G0016 | D04:336247..341449(-) | 729 | 9.00 | a,b,c,d | I | Plasma membrane |
| *GhCNGC22* | Gh_D04G0409 | D04:6457085..6464147(+) | 631 | 9.48 | a,b,c,d | IV-B | Plasma membrane |
| *GhCNGC23* | Gh_D04G0507 | D04:8647485..8650049(+) | 740 | 6.89 | a,b,c,d | III | Plasma membrane |
| *GhCNGC24* | Gh_D04G0818 | D04:18542044..18548380(-) | 731 | 9.29 | a,b,c,d | III | Plasma membrane |
| *GhCNGC25* | Gh_D05G0547 | D05:4438080..4442575(+) | 738 | 9.20 | a,b,c,d,e | II | Plasma membrane |
| *GhCNGC26* | Gh_D05G0985 | D05:8298111..8301011(-) | 710 | 9.18 | a,b,c,d | III | Plasma membrane |
| *GhCNGC27* | Gh_D05G1129 | D05:9678476..9682896(+) | 746 | 9.00 | a,b,c,d,e | II | Plasma membrane |
| *GhCNGC28* | Gh_D06G1113 | D06:25415590..25424220(-) | 885 | 9.47 | a,b,c,d | III | Plasma membrane |
| *GhCNGC29* | Gh_D07G1377 | D07:22261532..22264949(-) | 734 | 9.29 | a,b,c,d | I | Plasma membrane |
| *GhCNGC30* | Gh_D08G1044 | D08:29136722..29142273(-) | 753 | 8.83 | a,b,c,d | I | Plasma membrane |
| *GhCNGC31* | Gh_D09G0106 | D09:2641533..2647632(-) | 695 | 9.31 | a,b,c,d | IV-B | Plasma membrane |
| *GhCNGC32* | Gh_D09G0107 | D09:2667426..2672270(+) | 766 | 9.57 | a,b,c,d | IV-B | Plasma membrane |
| *GhCNGC33* | Gh_D09G1606 | D09:43634992..43640328(-) | 1057 | 9.06 | a,b,c,d | III | Plasma membrane |
| *GhCNGC34* | Gh_D11G0011 | D11:161114..174077(-) | 770 | 9.20 | a,b,c,d | IVA | Plasma membrane |
| *GhCNGC35* | Gh_D12G1124 | D12:38003265..38008325(+) | 719 | 9.51 | a,b,c,d | I | Plasma membrane |
| *GhCNGC36* | Gh_D13G1938 | D13:54757524..54759628(+) | 586 | 9.12 | a,b,c,d | III | Plasma membrane |
| *GhCNGC37* | Gh_D13G1974 | D13:55088065..55092625(+) | 743 | 8.97 | a,b,c,d,e | II | Plasma membrane |
| *GhCNGC38* | Gh_A05G3666 | scaffold1170_A05:953138..958831(+) | 738 | 9.34 | a,b,c,d | III | Plasma membrane |
| *GhCNGC39* | Gh_A09G2401 | scaffold2299_A09:87921..90512(+) | 692 | 9.06 | a,b,c,d | III | Plasma membrane |
| *GhCNGC40* | Gh_Sca006927G01 | scaffold6927:5643..9514(-) | 513 | 8.72 | a,b,c,d | I | Chloroplast |

Note: *Gossypium hirsutum* ($(AD)_1$) genome NAU-NBI Assembly v1.1 & Annotation v1.1.

Chr:Location(strand): chromosome & location (start..end) & strand.

AA: amino acids.

pI: isoelectric point.

D&M: domains & motif. a, transmembrane domains (TM); b, cyclic nucleotide-binding domain (CNBD) (InterPro: IPR000595, IPR018490); c, ion transport domain (ITP) (InterPro: IPR005821); d, plant CNGC-specific motif spanning the putative phosphate binding cassette (PBC) and the hinge within the CNBD (Zelman et al. *Front Plant Sci*. 2012, 3:95); e, IQ motif, EF-hand binding site (InterPro: IPR000048).

Group: phylogenetic classification in Figure 1.

**Table S3.** List of the CNGC family genes in *Gossypium barbadense*.

| Gene Name | Gene ID | Chr:Location(strand) | AA | pI | D&M | Group | Subcellular Localization |
|---|---|---|---|---|---|---|---|
| *GbCNGC1* | Gbar_A01G006020 | A01:8697293..8705568(+) | 709 | 8.76 | a,b,c,d | IV-B | Plasma membrane |
| *GbCNGC2* | Gbar_A01G021190 | A01:113941390..113944341(+) | 698 | 8.98 | a,b,c,d | III | Plasma membrane |
| *GbCNGC3* | Gbar_A03G003890 | A03:5102061..5105287(+) | 686 | 9.35 | a,b,c,d | III | Plasma membrane |
| *GbCNGC4* | Gbar_A05G005290 | A05:5025709..5027152(-) | 313 | 9.59 | b,d,e | II | Nuclear |
| *GbCNGC5* | Gbar_A05G010100 | A05:9148299..9152654(-) | 713 | 9.33 | a,b,c,d | III | Plasma membrane |
| *GbCNGC6* | Gbar_A05G034890 | A05:83328986..83336012(+) | 731 | 9.63 | a,b,c,d | III | Plasma membrane |
| *GbCNGC7* | Gbar_A05G036490 | A05:91999822..92002717(-) | 719 | 7.64 | a,b,c,d | III | Plasma membrane |
| *GbCNGC8* | Gbar_A05G037420 | A05:94694789..94698128(-) | 695 | 9.45 | a,b,c,d | IV-B | Plasma membrane |
| *GbCNGC9* | Gbar_A05G041690 | A05:102314038..102319578(+) | 712 | 9.13 | a,b,c,d | I | Plasma membrane |
| *GbCNGC10* | Gbar_A06G011120 | A06:42300192..42307280(-) | 709 | 9.11 | a,b,c,d | III | Plasma membrane |
| *GbCNGC11* | Gbar_A07G014600 | A07:29744624..29748343(-) | 718 | 9.77 | a,b,c,d,e | I | Plasma membrane |
| *GbCNGC12* | Gbar_A08G010210 | A08:49311693..49318179(-) | 708 | 9.16 | a,b,c,d | I | Plasma membrane |
| *GbCNGC13* | Gbar_A09G001260 | A09:2601895..2607487(-) | 688 | 9.34 | a,b,c,d | IV-B | Plasma membrane |
| *GbCNGC14* | Gbar_A09G001270 | A09:2635374..2641554(+) | 766 | 9.90 | a,b,c,d | IV-B | Plasma membrane |
| *GbCNGC15* | Gbar_A09G018060 | A09:70042981..70046031(-) | 692 | 9.01 | a,b,c,d | III | Plasma membrane |
| *GbCNGC16* | Gbar_A12G012100 | A12:74687268..74693244(+) | 708 | 10.06 | a,b,c,d,e | I | Plasma membrane |
| *GbCNGC17* | Gbar_A13G019770 | A13:103498649..103502436(+) | 727 | 9.17 | a,b,c,d | III | Plasma membrane |
| *GbCNGC18* | Gbar_A13G020140 | A13:103833121..103839357(+) | 743 | 9.04 | a,b,c,d,e | II | Plasma membrane |
| *GbCNGC19* | Gbar_D01G006380 | D01:8215486..8223064(+) | 709 | 8.80 | a,b,c,d | IV-B | Plasma membrane |
| *GbCNGC20* | Gbar_D03G014500 | D03:46018646..46022630(-) | 708 | 9.28 | a,b,c,d | III | Plasma membrane |
| *GbCNGC21* | Gbar_D04G000200 | D04:262035..268826(-) | 712 | 9.13 | a,b,c,d | I | Plasma membrane |
| *GbCNGC22* | Gbar_D04G004780 | D04:6645898..6649036(+) | 679 | 9.71 | a,b,c,d | IV-B | Plasma membrane |
| *GbCNGC23* | Gbar_D04G005720 | D04:8764155..8767091(+) | 714 | 6.96 | a,b,c,d | III | Plasma membrane |
| *GbCNGC24* | Gbar_D04G009040 | D04:18371339..18378487(-) | 731 | 9.57 | a,b,c,d | III | Plasma membrane |
| *GbCNGC25* | Gbar_D05G005530 | D05:4464122..4470130(+) | 736 | 9.30 | a,b,c,d,e | II | Plasma membrane |
| *GbCNGC26* | Gbar_D05G010670 | D05:8644929..8649959(-) | 713 | 9.26 | a,b,c,d | III | Plasma membrane |
| *GbCNGC27* | Gbar_D05G012040 | D05:10081285..10086992(+) | 746 | 9.06 | a,b,c,d,e | II | Plasma membrane |
| *GbCNGC28* | Gbar_D06G011530 | D06:24826696..24831210(-) | 588 | 9.24 | a,b,c,d | III | Plasma membrane |
| *GbCNGC29* | Gbar_D07G015050 | D07:22779814..22782638(-) | 712 | 9.71 | a,b,c,d,e | I | Plasma membrane |
| *GbCNGC30* | Gbar_D08G011030 | D08:30426796..30433111(+) | 708 | 9.22 | a,b,c,d | I | Plasma membrane |
| *GbCNGC31* | Gbar_D09G001180 | D09:2506003..2512155(-) | 688 | 9.40 | a,b,c,d | IV-B | Plasma membrane |
| *GbCNGC32* | Gbar_D09G001190 | D09:2531299..2536464(+) | 694 | 9.71 | a,b,c,d | IV-B | Plasma membrane |
| *GbCNGC33* | Gbar_D09G017790 | D09:44186045..44188613(-) | 637 | 8.90 | a,b,d | III | Plasma membrane |
| *GbCNGC34* | Gbar_D11G000120 | D11:131979..146215(-) | 770 | 9.24 | a,b,c,d | IV-A | Plasma membrane |
| *GbCNGC35* | Gbar_D12G012180 | D12:37968793..37974717(+) | 719 | 10.04 | a,b,c,d,e | I | Plasma membrane |
| *GbCNGC36* | Gbar_D13G019920 | D13:54336903..54339837(+) | 560 | 9.33 | a,b,c,d | III | Plasma membrane |
| *GbCNGC37* | Gbar_D13G020320 | D13:54643343..54649570(+) | 743 | 8.96 | a,b,c,d,e | II | Plasma membrane |
| *GbCNGC38* | Gbar_D01G024300 | Scaffold2826:49768..52446(+) | 706 | 8.81 | a,b,c,d | III | Plasma membrane |
| *GbCNGC39* | Gbar_D11G035610 | Scaffold2800:97663..116444(+) | 738 | 9.24 | a,b,c,d | IV-A | Plasma membrane |
| *GbCNGC40* | Gbar_Scaffold3324G000020 | Scaffold3324:18011..22438(+) | 748 | 8.89 | a,b,c,d,e | II | Plasma membrane |
| *GbCNGC41* | Gbar_Scaffold863G000020 | Scaffold863:4569..8992(+) | 748 | 8.89 | a,b,c,d,e | II | Plasma membrane |

Note: *Gossypium barbadense* ($(AD)_2$) genome HAU Assembly v2.0 & Annotation v1.0.

Chr:Location(strand): chromosome & location (start..end) & strand.
AA: amino acids.
pI: isoelectric point.
D&M: domains & motif. a, transmembrane domains (TM); b, cyclic nucleotide-binding domain (CNBD) (InterPro: IPR000595, IPR018490); c, ion transport domain (ITP) (InterPro: IPR005821); d, plant CNGC-specific motif spanning the putative phosphate binding cassette (PBC) and the hinge within the CNBD (Zelman et al. *Front Plant Sci*. 2012, 3:95); e, IQ motif, EF-hand binding site (InterPro: IPR000048).
Group: phylogenetic classification in Figure 1.

**Table S4.** List of the CNGC family genes in *Gossypium arboretum.*

| Gene Name | Gene ID | Chr:Location(strand) | AA | pI | D&M | Group | Subcellular Localization |
|---|---|---|---|---|---|---|---|
| *GaCNGC1* | Ga01G0732 | Chr01:9914558..9921711(+) | 709 | 8.93 | a,b,c,d | IVB | Plasma membrane |
| *GaCNGC2* | Ga01G2411 | Chr01:107207852..107210607(+) | 708 | 9.16 | a,b,c,d | III | Plasma membrane |
| *GaCNGC3* | Ga02G1657 | Chr02:97749586..97752188(+) | 715 | 8.96 | a,b,c,d | III | Plasma membrane |
| *GaCNGC4* | Ga04G0852 | Chr04:23568369..23572259(-) | 709 | 9.21 | a,b,c,d,e | I | Plasma membrane |
| *GaCNGC5* | Ga04G1517 | Chr04:86898684..86901259(-) | 717 | 7.52 | a,b,c,d | III | Plasma membrane |
| *GaCNGC6* | Ga04G1626 | Chr04:90143417..90146638(-) | 695 | 9.37 | a,b,c,d | IVB | Plasma membrane |
| *GaCNGC7* | Ga04G2110 | Chr04:98071052..98076588(+) | 712 | 9.10 | a,b,c,d | I | Plasma membrane |
| *GaCNGC8* | Ga05G0572 | Chr05:5145230..5149709(+) | 738 | 9.11 | a,b,c,d,e | II | Plasma membrane |
| *GaCNGC9* | Ga05G1104 | Chr05:9506677..9509703(-) | 713 | 9.19 | a,b,c,d | III | Plasma membrane |
| *GaCNGC10* | Ga05G1255 | Chr05:11108037..11112466(+) | 748 | 8.90 | a,b,c,d,e | II | Plasma membrane |
| *GaCNGC11* | Ga06G1223 | Chr06:47404847..47410039(-) | 714 | 8.93 | a,b,c,d | III | Plasma membrane |
| *GaCNGC12* | Ga08G1175 | Chr08:65285350..65290913(-) | 753 | 8.89 | a,b,c,d | I | Plasma membrane |
| *GaCNGC13* | Ga09G0093 | Chr09:2006369..2013520(+) | 712 | 9.45 | a,b,c,d | IVB | Plasma membrane |
| *GaCNGC14* | Ga09G0167 | Chr09:3622561..3627663(-) | 695 | 9.22 | a,b,c,d | IVB | Plasma membrane |
| *GaCNGC15* | Ga09G1844 | Chr09:76172097..76174688(-) | 692 | 9.06 | a,b,c,d | III | Plasma membrane |
| *GaCNGC16* | Ga11G4114 | Chr11:124328652..124350352(+) | 770 | 9.22 | a,b,c,d | IVA | Plasma membrane |
| *GaCNGC17* | Ga12G1804 | Chr12:29109060..29114159(-) | 708 | 9.58 | a,b,c,d,e | I | Plasma membrane |
| *GaCNGC18* | Ga13G2256 | Chr13:117824620..117828265(+) | 705 | 9.05 | a,b,c,d | III | Plasma membrane |
| *GaCNGC19* | Ga13G2293 | Chr13:118190385..118194964(+) | 743 | 9.06 | a,b,c,d,e | II | Plasma membrane |
| *GaCNGC20* | Ga14G2437 | Tig00021276:18324..24625(+) | 731 | 9.32 | a,b,c,d | III | Plasma membrane |

Note: *Gossypium arboreum* ($A_2$) genome CRI Assembly v1.0 & Annotation v1.0.

Chr:Location(strand): chromosome & location (start..end) & strand.

AA: amino acids.

pI: isoelectric point.

D&M: domains & motif. a, transmembrane domains (TM); b, cyclic nucleotide-binding domain (CNBD) (InterPro: IPR000595, IPR018490); c, ion transport domain (ITP) (InterPro: IPR005821); d, plant CNGC-specific motif spanning the putative phosphate binding cassette (PBC) and the hinge within the CNBD (Zelman et al. *Front Plant Sci*. 2012, 3:95); e, IQ motif, EF-hand binding site (InterPro: IPR000048).

Group: phylogenetic classification in Figure 1.

**Table S5.** List of the CNGC family genes in *Gossypium herbaceum.*

| Gene Name | Gene ID | Chr:Location(strand) | AA | pI | D&M | Group | Subcellular Localization |
|---|---|---|---|---|---|---|---|
| *GheCNGC1* | Ghe01G07250 | Chr01:8894127..8901253(+) | 709 | 8.73 | a,b,c,d | IV-B | Plasma membrane |
| *GheCNGC2* | Ghe01G27300 | Chr01:121183440..121185977(+) | 627 | 8.62 | a,b,c,d | III | Plasma membrane |
| *GheCNGC3* | Ghe02G04280 | Chr02:5055948..5058703(+) | 496 | 8.23 | a,b,c,d | III | Chloroplast |
| *GheCNGC4* | Ghe04G00530 | Chr04:619194..620813(-) | 313 | 9.66 | b,d | I | Mitochondrial inner membrane |
| *GheCNGC5* | Ghe04G05860 | Chr04:8878635..8881856(+) | 695 | 6.20 | a,b,c,d | IV-B | Plasma membrane |
| *GheCNGC6* | Ghe04G06950 | Chr04:11684349..11686924(+) | 579 | 6.20 | a,b,c,d | III | Chloroplast |
| *GheCNGC7* | Ghe04G10960 | Chr04:29417675..29423996(-) | 394 | 9.10 | a,b,d | III | Chloroplast |
| *GheCNGC8* | Ghe05G05920 | Chr05:5479046..5495297(+) | 1445 | 7.50 | a,b,c,d | II | Plasma membrane |
| *GheCNGC9* | Ghe05G11250 | Chr05:10302915..10305938(-) | 510 | 8.66 | a,b,c,d | III | Chloroplast |
| *GheCNGC10* | Ghe05G12750 | Chr05:11861550..11865985(+) | 713 | 8.54 | a,b,c,d,e | II | Plasma membrane |
| *GheCNGC11* | Ghe06G13600 | Chr06:46711807..46713030(-) | 348 | 9.60 | a,b,d | III | Chloroplast |
| *GheCNGC12* | Ghe07G16730 | Chr07:31265588..31268395(-) | 517 | 9.59 | a,b,c,d,e | I | Mitochondrial inner membrane |
| *GheCNGC13* | Ghe08G12560 | Chr08:55213223..55361119(-) | 524 | 7.42 | a,b,c,d | I | Chloroplast |
| *GheCNGC14* | Ghe09G01380 | Chr09:2706335..2713663(-) | 714 | 8.80 | a,b,c,d | IV-B | Plasma membrane |
| *GheCNGC15* | Ghe09G01390 | Chr09:2758562..2764089(+) | 686 | 9.99 | a,b,c,d | IV-B | Plasma membrane |
| *GheCNGC16* | Ghe09G21870 | Chr09:76207315..76208906(-) | 446 | 8.16 | a,b,c,d | III | Chloroplast |
| *GheCNGC17* | Ghe11G00200 | Chr11:208294..230215(-) | 786 | 8.42 | a,b,c,d | IV-A | Plasma membrane |
| *GheCNGC18* | Ghe12G15190 | Chr12:80976907..80982067(+) | 731 | 10.11 | a,b,c,d,e | I | Plasma membrane |
| *GheCNGC19* | Ghe13G24840 | Chr13:111635813..111637449(+) | 355 | 9.28 | a,b,d | III | Chloroplast |
| *GheCNGC20* | Ghe13G25210 | Chr13:111969677..111974253(+) | 541 | 8.62 | a,b,c,d,e | II | Nuclear |

Note: *Gossypium herbaceum* ($A_1$) genome WHU Assembly v1.0 & Annotation v1.0.

Chr:Location(strand): chromosome & location (start..end) & strand.

AA: amino acids.

pI: isoelectric point.

D&M: domains & motif. a, transmembrane domains (TM); b, cyclic nucleotide-binding domain (CNBD) (InterPro: IPR000595, IPR018490); c, ion transport domain (ITP) (InterPro: IPR005821); d, plant CNGC-specific motif spanning the putative phosphate binding cassette (PBC) and the hinge within the CNBD (Zelman et al. *Front Plant Sci*. 2012, 3:95); e, IQ motif, EF-hand binding site (InterPro: IPR000048).

Group: phylogenetic classification in Figure 1.

**Table S6.** List of the CNGC family genes in *Gossypium raimondii*

| Gene name | Gene ID | Chr:Location(strand) | AA | pI | D&M[$] | Group | Subcellular localization |
|---|---|---|---|---|---|---|---|
| *GrCNGC1* | Gorai.001G155300 | Chr01:21774055..21777693(-) | 712 | 9.34 | a,b,c,d,e | I | Plasma membrane |
| *GrCNGC2* | Gorai.002G076600 | Chr02:9066932..9074583(+) | 709 | 9.02 | a,b,c,d | IVB | Plasma membrane |
| *GrCNGC3* | Gorai.002G248200 | Chr02:61219773..61222311(+) | 698 | 9.10 | a,b,c,d | III | Plasma membrane |
| *GrCNGC4* | Gorai.003G143300 | Chr03:40840047..40843299(-) | 708 | 9.16 | a,b,c,d | III | Plasma membrane |
| *GrCNGC5* | Gorai.004G115300 | Chr04:27570395..27576917(-) | 582 | 8.78 | a,b,c,d | I | Plasma membrane |
| *GrCNGC6* | Gorai.006G012500 | Chr06:2655669..2661881(-) | 688 | 9.34 | a,b,c,d | IVB | Plasma membrane |
| *GrCNGC7* | Gorai.006G012600 | Chr06:2689723..2695126(+) | 766 | 9.53 | a,b,c,d | IVB | Plasma membrane |
| *GrCNGC8* | Gorai.006G187100 | Chr06:44397567..44400915(-) | 692 | 9.07 | a,b,c,d | III | Plasma membrane |
| *GrCNGC9* | Gorai.007G002000 | Chr07:201004..215206(-) | 770 | 9.29 | a,b,c,d | IVA | Plasma membrane |
| *GrCNGC10* | Gorai.008G124800 | Chr08:36428101..36434147(+) | 719 | 9.54 | a,b,c,d,e | I | Plasma membrane |
| *GrCNGC11* | Gorai.009G056700 | Chr09:4097678..4103911(+) | 745 | 9.19 | a,b,c,d,e | II | Plasma membrane |
| *GrCNGC12* | Gorai.009G108500 | Chr09:7879763..7884030(-) | 715 | 9.21 | a,b,c,d | III | Plasma membrane |
| *GrCNGC13* | Gorai.009G123300 | Chr09:9200673..9206164(+) | 746 | 9.00 | a,b,c,d,e | II | Plasma membrane |
| *GrCNGC14* | Gorai.010G121000 | Chr10:24503137..24509026(-) | 709 | 9.13 | a,b,c,d | III | Plasma membrane |
| *GrCNGC15* | Gorai.010G245300 | Chr10:61428605..61431508(-) | 641 | 9.25 | a,b,c,d | III | Plasma membrane |
| *GrCNGC16* | Gorai.012G003500 | Chr12:416603..423954(-) | 712 | 9.10 | a,b,c,d | I | Plasma membrane |
| *GrCNGC17* | Gorai.012G050600 | Chr12:6577438..6580915(+) | 695 | 9.41 | a,b,c,d | IVB | Plasma membrane |
| *GrCNGC18* | Gorai.012G060800 | Chr12:8516862..8519426(+) | 714 | 6.53 | a,b,c,d | III | Plasma membrane |
| *GrCNGC19* | Gorai.012G095800 | Chr12:17638792..17646002(-) | 738 | 9.25 | a,b,c,d | III | Plasma membrane |
| *GrCNGC20* | Gorai.013G215800 | Chr13:53579111..53585314(+) | 743 | 9.01 | a,b,c,d,e | II | Plasma membrane |

Note: *Gossypium raimondii* ($D_5$) genome JGI Assembly v2.0 & Annotation v2.1.

Chr:Location(strand): chromosome & location (start..end) & strand.

AA: amino acids.

pI: isoelectric point.

D&M: domains & motif. a, transmembrane domains (TM); b, cyclic nucleotide-binding domain (CNBD) (InterPro: IPR000595, IPR018490); c, ion transport domain (ITP) (InterPro: IPR005821); d, plant CNGC-specific motif spanning the putative phosphate binding cassette (PBC) and the hinge within the CNBD (Zelman et al. *Front Plant Sci*. 2012, 3:95); e, IQ motif, EF-hand binding site (InterPro: IPR000048).

Group: phylogenetic classification in Figure 1.
